# The many faces of Dicer: the complexity of the mechanisms regulating Dicer gene expression and enzyme activities

**DOI:** 10.1093/nar/gkv328

**Published:** 2015-04-16

**Authors:** Anna Kurzynska-Kokorniak, Natalia Koralewska, Maria Pokornowska, Anna Urbanowicz, Aleksander Tworak, Agnieszka Mickiewicz, Marek Figlerowicz

**Affiliations:** 1 Institute of Bioorganic Chemistry, Polish Academy of Sciences, Poznan 61-704, Poland; 2 Institute of Computing Science, Poznan University of Technology, Poznan 60-965, Poland

## Abstract

There is increasing evidence indicating that the production of small regulatory RNAs is not the only process in which ribonuclease Dicer can participate. For example, it has been demonstrated that this enzyme is also involved in chromatin structure remodelling, inflammation and apoptotic DNA degradation. Moreover, it has become increasingly clear that cellular transcript and protein levels of Dicer must be strictly controlled because even small changes in their accumulation can initiate various pathological processes, including carcinogenesis. Accordingly, in recent years, a number of studies have been performed to identify the factors regulating Dicer gene expression and protein activity. As a result, a large amount of complex and often contradictory data has been generated. None of these data have been subjected to an exhaustive review or critical discussion. This review attempts to fill this gap by summarizing the current knowledge of factors that regulate Dicer gene transcription, primary transcript processing, mRNA translation and enzyme activity. Because of the high complexity of this topic, this review mainly concentrates on human Dicer. This review also focuses on an additional regulatory layer of Dicer activity involving the interactions of protein and RNA factors with Dicer substrates.

## INTRODUCTION

Small regulatory RNAs, such as microRNAs (miRNAs) or small interfering RNAs (siRNAs), play essential roles in many important biological processes, including developmental timing, growth control, differentiation and apoptosis (1–3). In humans, the vast majority of small regulatory RNAs are miRNAs. To date, ∼2600 miRNAs encoded in the human genome that are derived from almost 1900 miRNA precursors (pre-miRNAs) have been identified (http://www.mirbase.org/). They have been found to control the expression of most human protein-coding genes through the miRNA pathway (4,5). Moreover, it has been demonstrated that miRNAs play a very important role in host-virus interactions in mammals (6–9). Therefore, the cellular levels of miRNAs and other components of miRNA pathways must be tightly controlled, both spatially and temporally. Aberrant regulation of miRNA levels can initiate pathological processes, including carcinogenesis as well as neurodegenerative, immune system and rheumatic disorders (10–13).

A fundamental role in the biogenesis of miRNAs in humans is played by a ribonuclease III (RNase III) enzyme termed Dicer, which recognizes and cleaves 50–70-nucleotide (nt) single-stranded pre-miRNAs with hairpin structures or double-stranded RNAs (dsRNAs) into functional 21–23-nt miRNAs or siRNAs, respectively (14). Human Dicer is a 220-kDa multidomain enzyme comprising an amino (N)-terminal putative helicase domain (homologous to DExD/H-box helicases), a DUF283 domain (domain of unknown function), a PAZ (Piwi-Argonaute-Zwille) domain, two RNase III domains (RNase IIIa and RNase IIIb) and a dsRNA-binding domain (dsRBD) (14–18). Because all metazoan Dicers are large and complex proteins, they are difficult to crystallize. A lack of structural data has limited the understanding of Dicer-mediated processes. The crystal structure of an intact Dicer enzyme has only been determined for *Giardia intestinalis*; however, *Giardia* Dicer is only comprised of the PAZ and tandem RNase III domains and lacks many of the domains and regions characteristic of Dicers in higher eukaryotes (16). A three-dimensional model of the human Dicer enzyme has been determined based on electron microscopy data (19–22). In addition, structures of several individual Dicer domains have been established, including the crystal structures of the platform-PAZ-connector helix cassette (23) and the carboxy (C)-terminal RNase III domain (RNase IIIb) (24). Moreover, a three-dimensional model of the DUF283 domain has been generated by computational methods (25).

The first model of Dicer ribonuclease activity was proposed in 2004 by Filipowicz *et* *al*. (18). According to this model, miRNA and siRNA precursors are recognized by the PAZ domain. The latter binds to the 3′ end of the substrate, with a preference for 2-nt-long overhangs (18,26–29). The dsRBD of Dicer has been shown to play only an auxiliary role in substrate binding and cleavage. This model also implies that Dicer contains a single dsRNA cleavage centre formed by the RNase IIIa and RNase IIIb domains, which are both located within the same molecule. This enzyme cuts both strands of dsRNA precursors at regions located ∼20 base pairs (bp) from their termini (18). This model has been further improved by the enhanced identification and characterization of the functions of other Dicer domains and motifs. Currently, it is assumed that Dicer has two pockets that bind to substrate ends, a 3′-end-binding pocket located within the PAZ domain (16,18) and a 5′-end-binding pocket located within the PAZ domain and the so-called platform domain (23,30). Importantly, docking to the 5′-end-binding pocket has been proposed to be efficient only when substrate ends are less stably base-paired, which is more characteristic of miRNA precursors (23). Accordingly, analyses of Dicer homologues have revealed that the 5′-end-binding pocket is highly conserved among most Dicers producing miRNAs but not siRNAs. Thus, this motif is not present in Dicers from lower eukaryotes (e.g. *Giardia* and fungi), which lack the miRNA pathway (30). Furthermore, recent data have suggested that the N-terminal helicase domain of Dicer is involved in the discrimination between miRNA and siRNA precursors by interacting with the hairpin loop structures of pre-miRNAs (21,31–33). Importantly, RNA binding by the helicase domain has been proposed to cause substrate-dependent changes in the Dicer structure (21,34). Nevertheless, the helicase domain has also been shown to be dispensable for Dicer cleavage activity (35,36). Further, this domain has been proposed to function as an autoinhibitor of Dicer (37). The function of the DUF283 domain remains unknown. Initially, it was suggested to be critical for pre-miRNA processing because Dicer mutants lacking it (in addition to the helicase domain) lost this activity (35,36). However, Doudna *et* *al*. have shown that the cleavage activity of a Dicer mutant with a deletion of the DUF283 domain, but possessing all other components, is only slightly affected (37). Individual Dicer domains have also been shown to interact with other proteins. This issue will be discussed later in this manuscript.

Based on the first models of Dicer proteins it was proposed that the length of the small RNAs produced by Dicer was determined by the distance between the PAZ domain and the cleavage centre, which depends on the length of the linker that connects them (16,19). Thus, Dicer was considered to be a molecular ruler that measured and cleaved 20–25-bp duplexes from dsRNA substrates. Interestingly, Doudna *et* *al*. have recently shown that active human Dicer can be reconstituted from two or three separately obtained fragments (31). More importantly, the two fragments, one comprising the DUF283 and PAZ domains (N-terminal) and the other comprising the RNase III and dsRBD domains (C-terminal), could still produce 22-bp products, although both fragments lacked a portion of the linker connecting the PAZ and RNase III domains (31). Thus, one can hypothesize that the lengths of Dicer-generated products are determined by the nature of PAZ and RNase III domain interactions, which may occur either directly or through an RNA substrate.

The ribonuclease Dicer is found throughout eukaryotes but is absent in bacteria and archaea. It has been suggested that the Dicer family has independently diversified in animal, plant and fungal lineages (38,39); however, it has been lost from some protozoan parasites (e.g. *Leishmania major* and *Trypanosoma cruzi*) and some fungi (e.g. the model organism *Saccharomyces cerevisiae* and other closely related yeasts) (40,41). Current evidence suggests that the Dicer gene underwent duplication early during animal and plant evolution, presumably coinciding with the origin of multicellularity, giving rise to two distinct groups in animals (Dicer-1 and Dicer-2) and to four groups in plants (Dicer-like (DCL) proteins DCL-1 to DCL-4). In insects, Dicer-1 and Dicer-2 have been shown to recognize distinct substrates and to generate different classes of small RNAs; i.e. miRNAs and siRNAs, respectively (42). Furthermore, Dicer-2 has been shown to cleave cell-derived dsRNA precursors to produce endogenous siRNAs (43), in addition to double-stranded virus-derived RNAs to produce exogenous siRNAs (44). Accordingly, an essential function of Dicer-2 in host defense against RNA viruses has been documented for *Drosophila* (44). Interestingly, Dicer-2 was subsequently lost from lineages that developed alternative antiviral strategies, such as vertebrates (39).

Since its discovery in 2000 (14), the ribonuclease Dicer has been considered to be one of the key factors responsible for the production of small regulatory RNAs. Consequently, a number of articles thoroughly discussing various aspects of Dicer involvement in the biogenesis of miRNAs and siRNAs have been published. However, an overall understanding of the gene expression and protein functions of this enzyme is still far from being elucidated. Thus, this review summarizes the current knowledge of various factors involved in the regulation of Dicer gene transcription, primary transcript processing, mRNA translation and enzyme activity. Because of the high complexity of the topics that are discussed, we mainly focus on human Dicer. Finally, as mutations in the Dicer gene may generate a range of phenotypes that are correlated with many types of cancer, we also discuss the association of Dicer with cancer.

## ORGANIZATION OF THE DICER GENE AND FACTORS CONTROLLING ITS EXPRESSION

**Figure 1.**
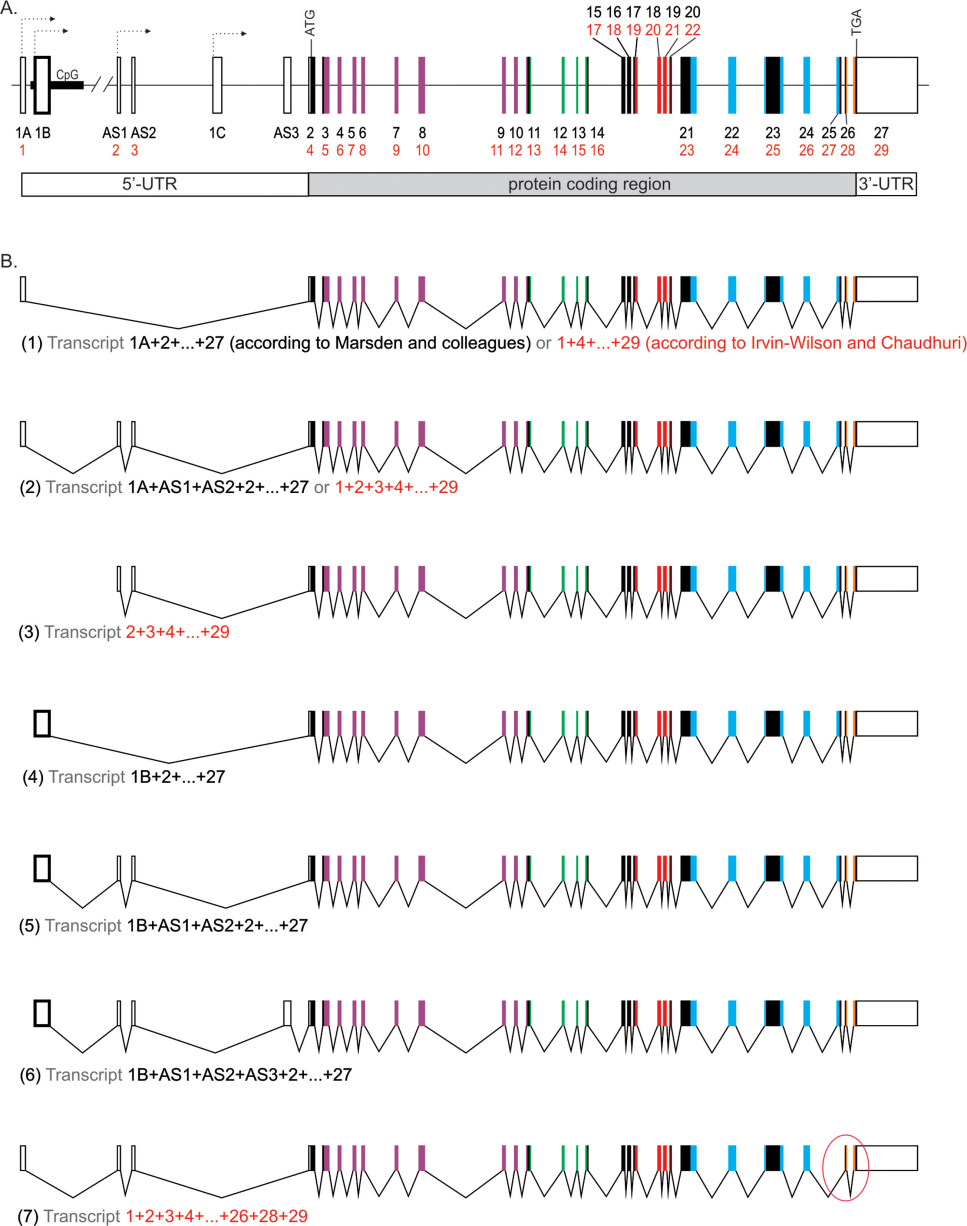
Schematic diagram of (**A**) the organization of the human Dicer gene and (**B**) exemplary Dicer transcript variants. The nomenclature of exons adopted by Marsden *et al.* (45) is indicated in black and that adopted by Irvin-Wilson and Chaudhuri (46) is indicated in red. The non-protein-coding exons, indicated by open boxes, form the 5′-UTR and are described by Marsden *et al.* as variants of exon 1 (leader exons 1A, 1B and 1C; and alternatively spliced exons abbreviated AS—AS1, AS2 and AS3), while those described by Irvin-Wilson and Chaudhuri are numbered 1, 2 and 3. The alternative transcription initiation sites are indicated by arrows. Exon 1B is boxed in bold and is located within a genomic sequence containing a CpG island. Fragments of exons coloured in purple encode a helicase domain, whereas the green fragments encode a DUF283 domain, the red fragments encode a PAZ domain, the blue fragments encode both RNase IIIa and RNase IIIb domains and the orange fragments encode a dsRBD domain. The transcript variant no. (7), for which one of the protein-coding exons is skipped (the region circled in red), encodes a Dicer protein lacking the majority of its RNase IIIb domain. A detailed description can be found in the text.

In all vertebrates, only one gene encoding the Dicer protein has been identified; however, its chromosomal location, as well as the number of exons it contains, varies between species. The human Dicer-coding gene *(DICER1)* is located on chromosome 14 and contains 26 protein-coding exons and a few non-protein-coding exons. The latter form the 5′-untranslated region (5′-UTR); (http://www.ensembl.org/Homo_sapiens/Gene/Summary?g=ENSG00000100697;r=14:95552565--95624347). The organization of *DICER1* is presented in Figure 1A. The dominant initiation codon (AUG) is found within exon 2 according to the nomenclature of exons adopted by Marsden *et* *al*. (indicated in black in Figure 1) (45) and it is found within exon 4 according to the nomenclature adopted by Irvin-Wilson and Chaudhuri (indicated in red in Figure 1) (46).

Many factors can regulate the transcription of *DICER1*. These factors can be universal or cell-, tissue- or stage-specific. For instance, SOX4, a transcription factor involved in the regulation of embryonic development and in the determination of cell fate, positively regulates the expression of the Dicer gene by binding to its promoter and enhancing its activity (47). SOX4 is also a well-known oncogene, and its overexpression is observed in many types of cancers (48–50), including prostate cancer (51). Accordingly, in human prostate cancer cells, the level of Dicer mRNA has been found to be upregulated (52). Another report on the transcriptional regulation of Dicer gene expression has shown that upon melanocyte differentiation, the melanocyte master transcriptional regulator, microphthalmia-associated transcription factor (MITF), binds to and activates a conserved regulatory sequence located upstream of the transcriptional start site of the Dicer gene, thereby stimulating its expression in these cells (53). It is also not surprising that within the promoter of the Dicer gene, target sequences for the ubiquitous tumour suppressors p53 and p63 have been identified (54). Nevertheless, the current knowledge of transcription factors influencing the expression of the Dicer gene is still very limited. The complexity of this process is highlighted by the fact that the level of Dicer transcripts is not always correlated with its protein level, implying that the regulation of its expression may occur at the post-transcriptional level (55). For example, Wiesen and Tomasi have used different human and mouse cell lines to demonstrate that histone deacetylase inhibitors only modestly alter Dicer mRNA levels but substantially decrease its protein levels, possibly by activating cellular stress-response pathways (55). These authors have also shown that at the protein level, Dicer expression may be downregulated by ∼4000 bp-long dsRNAs and interferon alpha (type I interferon) and upregulated by interferon gamma (type II interferon).

Currently, it is clear that from one Dicer gene, multiple Dicer transcript variants can be produced as a result of the initiation of transcription from alternative promoters and alternative splicing (Figure 1B). Four mRNA variants that encode full-length human Dicers (comprising 1922-amino acid residues) have been identified to date (http://www.ensembl.org/Homo_sapiens/Gene/Summary?g=ENSG00000100697;r=14:95552565--95624347). These four variants differ in their 5′ and 3′ non-protein-coding sequences, which contain different regulatory elements, while their coding regions remain unchanged. In addition, numerous shorter alternative splice variants have been found. Some of these splice variants encode proteins retaining only the N- or C-terminus of Dicer, while some variants do not encode any protein. Four antisense transcripts associated with *DICER1* have also been identified, ranging from ∼720 to 2300 nt in length (http://www.ensembl.org/Homo_sapiens/Transcript/Summary?db=core;g=ENSG00000235706;r=14:95643820--95646262;t=ENST00000439999). Their functions have not yet been established. However, it has recently been reported that antisense transcripts may regulate the activity of the genes from which they are derived (56,57).

Two research groups, Marsden *et* *al*., working with various healthy human tissues (45), and Irvin-Wilson and Chaudhuri, working with human breast cells (46), have extensively analysed the organization of the 5′-UTRs of Dicer mRNAs. Both of these groups have demonstrated that the observed diversity within these 5′-UTRs is associated with the tissue- and developmental-specific expression of the Dicer gene. Moreover, they have found that its organization impacts the translational efficiency of Dicer mRNA. The results of the very first studies of Dicer transcripts, which were conducted by Hamaguchi *et* *al*., suggested that many potential upstream open reading frames are present within the 5′-UTR (58). This issue was also assessed by Marsden *et* *al*. (45) and by Irvin-Wilson and Chaudhuri (46). The latter authors have reported that Dicer transcripts isolated from human breast cells have a high number of upstream start codons in-frame with stop codons. Such a composition of the 5′-UTR has been shown to decrease the stability of Dicer transcripts. These authors have also found that in healthy human breast cells, two predominant 5′-UTR variants of *DICER1* mRNA are produced (compare transcript no. (1) and (2) in Figure 1B). The shorter variant, which lacks the two exons with additional AUG codons (exons 2 and 3, according to the nomenclature adopted by Irvin-Wilson and Chaudhuri), has been shown to be more efficiently translated in an *in vitro* reporter system (46). These authors have also shown that in human breast cells, instead of the previously identified promoters (58), an alternative promoter located far upstream is employed for Dicer gene expression (46) (compare transcript nos. (1–2) and (3) in Figure 1B). In addition, Marsden *et* *al*. have identified unique 5′-UTRs formed by a combination of three leader exons (1A, 1B and 1C) and three alternatively spliced exons (AS1, AS2 and AS3) (Figure 1A and B). Furthermore, these authors have demonstrated *in vitro* that the presence of any of the three alternatively spliced exons (AS1, AS2 or AS3) in the 5′-UTR decreases the translation of the reporter gene. However, the lowest translation efficiency has been detected for transcripts containing the leader exon 1B, which is expressed from the alternative long-distance CpG island promoter (45) (transcript nos. (4–6) in Figure 1B; exon 1B is boxed in bold).

Reports of the alternative processing of human Dicer pre-mRNA, as discussed above, have also revealed that shorter Dicer mRNA variants are produced in differentiated epithelial cells and in a number of cancer cell lines (59,60). An interesting splice variant of the human Dicer gene has been identified in neuroblastoma cells (61). In this variant, one of the protein-coding exons is skipped and as a result, the reading frame is altered and a premature stop codon is gained (transcript no. (7); region circled in red in Figure 1B). Consequently, a protein that is 93 amino acid residues shorter than the full-length Dicer and differs in the last 41 C-terminal amino acids is produced (lacking one of the two RNase III domains and the dsRBD) (61). It is not clear whether this truncated protein is a hallmark of neuroblastoma development or whether it plays a role in differentiation and tumorigenesis. The influence of the dysfunction of a single RNase III domain on Dicer activity has been studied by Sharp *et* *al*. (62) and Amatruda *et* *al*. (63). These groups have observed that this enzyme can retain partial activity even if one RNase III domain is inactive. Specifically, Dicer with a dysfunctional RNase IIIa domain fails to produce miRNAs from the 3′-arm of pre-miRNA hairpins (62), whereas Dicer with an affected RNase IIIb domain does not produce miRNAs from the 5′-arm of pre-miRNA hairpins (62,63). Those results provide insights into the mechanisms by which mutations in Dicer may affect the level of miRNA expression and thus trigger tumorigenesis.

Some authors considered the Dicer gene to be a housekeeping gene (64); however, compared with other housekeeping genes, its 3′-UTR-encoding fragment has been shown to be unusually long (>4000 bp) (64). In Dicer mRNA, this region is involved in the post-transcriptional regulation of gene expression. The 3′-UTR of Dicer mRNA can be targeted by several miRNAs, e.g. miR-103/107 (59,60,64), miR-192 (65) or members of the let-7 miRNA family (60,66). Interestingly, target sequences for let-7 miRNAs have also been found within the coding regions of Dicer transcripts (66). Furthermore, Mayr and Bartel have identified various polyadenylation signals at the 3′ ends of Dicer pre-mRNAs in multiple cancer cell lines (67). These authors have shown that in cancer cells, the production of Dicer from a more abundant mRNA with a shorter 3′-UTR is several times greater than that from an mRNA with a longer 3′-UTR (67). Shorter mRNAs that lack large 3′-UTR fragments, which contain a number of miRNA-binding sites, cannot be targeted by RNA-silencing machinery. Thus, the regulation of the level of Dicer mRNA by at least some miRNAs is lost in cells carrying such shortened transcripts. The problem associated with the use of alternative polyadenylation sites within Dicer pre-mRNAs has also been discussed by Kanaoka *et al*. (68). These authors have demonstrated that in colorectal cancer, primary Dicer transcripts are polyadenylated at two alternative sites, giving rise to two transcript isoforms with either a long or truncated 3′-UTR (68).

Another report concerning Dicer transcripts has postulated that Dicer mRNA may specifically interact with exportin-5, a nuclear receptor involved in the export of certain classes of RNAs, including pre-miRNAs, viral hairpin RNAs and some tRNAs (69). All of these RNAs compete for binding to exportin-5. Thus, the saturation of this receptor with other RNAs decreases the export of Dicer mRNA to the cytoplasm and as a consequence, reduces further Dicer gene expression.

It has also been found that the level and/or activity of human Dicer can be regulated by its post-translational modification, e.g. phosphorylation (70) and SUMOylation (71). Relationships among phosphorylation, a major cell signalling pathway (the Ras pathway), and the core miRNA machinery have been previously demonstrated for a Dicer-interacting protein, the trans-activation response RNA binding protein (TRBP) (72). Very recently, Arur *et* *al*. have shown that Ras signalling also results in Dicer phosphorylation during oogenesis in *Caenorhabditis elegans* (73). These authors have demonstrated that extracellular signal-regulated kinase (ERK) phosphorylates Dicer (within the RNase IIIb and dsRBD domains) during most of oogenesis and that this phosphorylation is necessary and sufficient to trigger Dicer's nuclear translocation in worm, mouse and human cells (73). In addition to inducing nuclear localization, Dicer phosphorylation has also been found to inhibit its function (73). Interestingly, Dicer has been observed to be rapidly dephosphorylated just before fertilization (73).

Dicer also contains several potential lysine SUMOylation sites (71). It has been demonstrated that cigarette smoke may alter alveolar macrophage miRNA production by Dicer SUMOylation. The latter process has been found to decrease Dicer activity, resulting in the massive downregulation of miRNAs and presumably promoting smoking-related diseases (71). These findings demonstrate that environmental exposure may cause changes in miRNA levels via the post-translational modification of Dicer.

Dicer has also been proposed to be a glycoprotein (74). In addition to evidence that Dicer is translocated through the endoplasmic reticulum, it has been suggested that glycosylation of this enzyme may play a role in maintaining its intracellular level (74). The conjugation of carbohydrate chains to proteins may contribute to their proper folding; however, the functional implications of this modification in the case of Dicer remain elusive and require further investigation.

## DICER EXERTS ITS FUNCTIONS AS PART OF LARGE MULTIPROTEIN COMPLEXES

Although Dicer alone is capable of cleaving pre-miRNA and long dsRNA to miRNA and siRNA, respectively, its catalytic activity is known to be modulated by associated proteins, particularly by two closely related proteins, TRBP and protein activator of protein kinase R (PACT) (35,75,76). These two Dicer-binding proteins are important regulators that contribute both substrate and cleavage specificity during small regulatory RNA production. In particular, it has been reported that Dicer, in association with PACT, preferentially cleaves pre-miRNAs rather than precursors of siRNAs (77). This discrimination is less pronounced when Dicer is coupled with TRBP (77). These findings are consistent with those of another study, showing that *in vitro* PACT binds siRNA at a lower affinity than TRBP (78). Moreover, both proteins not only influence substrate discrimination by Dicer but also may change the cleavage site, thereby triggering the generation of different sized iso-miRNAs (isomiRs) (77,79). Additionally, the direct binding of TRBP to pre-miRNA substrates has been proposed to increase the initial rate of substrate recognition by Dicer (80). TRBP has also been shown to increase the stability of Dicer/substrate complexes and all of these components together presumably stimulate dicing (80). Furthermore, evidence indicates that TRBP, in association with Dicer, contributes not only to RNA substrate binding and product length determination (79–83) but also to the assembly of larger multiprotein complexes, including the RNA-induced silencing complex (RISC) and RISC-loading complex (RLC) (22,75,76,84). Similar roles have been proposed for PACT (35); however, its specific function is still poorly understood. RISC is a central effector of RNA-silencing pathways (85–87). The functional core of every RISC is composed of a member of the Argonaute (Ago) protein family and a small regulatory RNA (88,89). The latter guides the RISC to a target RNA transcript, permitting its binding through Watson–Crick base pairing (87). Maniataki and Mourelatos have proposed that Dicer, in association with Ago2, binds to and cleaves pre-miRNA. Next, the resultant miRNA duplex is passed along to Ago2 via the cooperation of Dicer and TRBP (90). In humans, Dicer, TRBP and Ago2 form the core of the RLC (84,87). Electron microscopy and single particle image analysis of a reconstituted RLC have revealed that within the ternary complex, the Dicer N-terminal helicase domain interacts with TRBP, while the C-terminal catalytic domains of RNase III are proximal to Ago2 (22). Interestingly, Doudna *et* *al*. have shown that *in vitro*, the duplex generated by Dicer cleavage may be released from the Dicer/TRBP complex and may rebind in a different orientation in the helicase domain of Dicer before it is loaded onto Ago2 (91). The fate of the RLC following the loading of Ago2 with duplex RNA is elusive. Some research groups have suggested that the complex remains intact (35,87) and that the presence of Dicer and TRBP stimulates target RNA processing by Ago2 (75,87). Other groups either have reported the dissociation of Dicer from Ago2 after the latter is loaded with an RNA duplex (90) or have assumed that Dicer does not participate directly in the slicer activity of the RISC (84). Thus, the role of Dicer in coupling miRNA biogenesis and post-transcriptional gene silencing remains unclear and requires further investigation. Notably, it has been suggested that Dicer may not be required for the assembly of siRNA-containing RISCs *in vivo* in mice (92) and *in vitro* in humans (93). Hence, it is possible that the mechanisms of RISC assembly in the miRNA and siRNA pathways are different.

It has also been demonstrated that RISC or RISC-like complexes can operate in nucleus (94,95). However, the data collected indicate that nuclear RISC is much smaller than the cytoplasmic RISC complex. The estimated size of the former one is close to the size of the single Argonaute protein (94). Thus, one can assume that the nuclear RISC complex lacks Dicer.

Another protein partner that can modulate the activity of Dicer is adenosine deaminase acting on RNA 1 (ADAR1), which catalyzes the adenosine-to-inosine editing of dsRNA and pri- and pre-miRNAs (34,96–98). According to current data, the editing activity of ADAR1 is attributed to its homodimer form, whereas heterodimers of ADAR1 and Dicer do not exhibit editing activity (96). It has been further demonstrated that ADAR1 forms a complex with Dicer through a direct protein–protein interaction involving the DUF283 and DEAD-box RNA helicase domains of Dicer (96). As mentioned above, the helicase domain has also been reported to be involved in interacting with TRBP (22,76,99), indicating that TRBP and ADAR1 associate with at least one common Dicer domain. In addition, it has been postulated that ADAR1 increases the rate of substrate cleavage by inducing conformational changes in Dicer (96).

## NON-miRNA-RELATED INTERACTION NETWORK OF DICER

Early studies of RNA interference (RNAi) phenomena and miRNA biogenesis pathways have led to the prevailing concept that Dicer is localized solely to the cytoplasm (100). In addition, several research groups have demonstrated that a small pool of Dicer co-purifies with membranes (74,76,101). More recently, it has been shown that mammalian Dicer can also function in the nucleus, where it has been found to be associated with nuclear ribosomal DNA (rDNA) chromatin, precisely interacting with the transcribed and promoter regions of rDNA repeats (102). Nevertheless, in mammals, the roles of these interactions remain unclear. It is possible that the association of Dicer with rDNA repeats preserves the stability of this region (102). Such a role of Dicer analogues has been previously shown in flies (103) and yeast (104). In various species, Dicer has also been demonstrated to link the RNAi pathway to heterochromatin assembly (105–111). According to the model proposed by Grewal *et* *al*. for yeast, nuclear Dicer generates siRNA associated with a nuclear Argonaute complex, termed the RNA-induced transcriptional silencing complex (RITS) (108,109). In addition to Argonaute proteins, the RITS contains histone-binding and adaptor proteins. It recruits histones and histone-modifying enzymes to target chromatin and generates repressed chromatin structures in a process called transcriptional gene silencing (108,109). Furthermore, it has been demonstrated in mammalian cells that a reduction in the Dicer level results in a more open chromatin structure due to a decrease in methylation, an increase in the acetylation of histones, and the loss of the chromatin-bound Argonaute proteins (112,113). Likewise, a depletion of Dicer in a human embryonic cell line has been shown to activate chromatin at the *PHLDA2* locus, an important tumour suppressor gene region (114). The observed phenomenon was linked to the changes in the level of histone acetylation but not to the methylation state of the locus (114). It was later suggested that human Dicer, under normal conditions, is recruited to loci of endogenous overlapping transcription through association with RNA polymerase II and dsRNA (110). Dicer then co-transcriptionally cleaves dsRNA into siRNA, leading to Ago1 recruitment (110). All of these events prevent endogenous dsRNA formation from overlapping non-coding RNA transcription units, precluding an uncontrolled interferon response and cellular apoptosis (110).

Interestingly, Argonaute and Dicer proteins have also been shown to be able to affect splicing (112,115). Most splicing events occur co-transcriptionally (116,117). Batsche *et* *al*. have proposed a model in which nuclear Argonaute proteins are guided to the vicinity of potential splice sites by Dicer-generated small RNAs interacting with intragenic antisense transcripts synthesized by RNA polymerase II. Argonaute proteins, in association with guiding RNAs, are recruited to the spliceosome complex through interactions with proteins bound to pre-mRNAs that are also produced by RNA polymerase II (112). As a result, transcriptase activity slows, facilitating spliceosome recruitment and inducing splicing events (112).

Recent studies have also revealed that Dicer is involved in the cell response to double-strand breaks (DSBs) in both animals and plants (118,119). Chromosome damage can occur, for example, due to oncogenic stress, ionizing radiation or the activities of site-specific endonucleases (118). In these situations, the efficient repair of DSBs is critical for the maintenance of genome integrity and cell survival. Dicer presumably processes RNAs formed by the transcription of broken DNA ends (120). It has been demonstrated in Arabidopsis and human cells that 21-nt small RNAs are produced from sequences flanking DSB sites (119). These small RNAs have been named diRNAs, which stands for DSB-induced small RNAs. It has been postulated that diRNAs function as guide molecules that direct chromatin modification or recruit protein complexes that facilitate DSB repair (119).

One recent report has also demonstrated that Dicer, together with PACT, TRBP and PKR, acts as a co-regulator of nuclear receptors (121), establishing a connection between core miRNA machinery and nuclear receptor signalling networks. These RISC components have been shown to interact with steroid receptor RNA activators, which recruit RISC proteins to steroid-responsive promoters, where they regulate the expression of downstream genes (121).

It is also important to mention that loss of Dicer in mouse oocytes has been shown to be associated with severe chromosome congression defects, presumably due to disorganized spindle formation (122,123). Moreover, Dicer depletion in mouse oocytes has been linked with upregulation of some retrotransposon families (122). Thus, Dicer seems to be a crucial player in the regulatory network that controls oocyte gene expression programs and integrity of the oocyte genome.

Dicer-interacting factors also include a group of viral proteins. Some RNA viruses, such as hepatitis C virus or human immunodeficiency virus, produce proteins that may inhibit the activity of Dicer; hence, these proteins have been termed viral suppressors of RNA silencing. These viral proteins may directly interact with Dicer (e.g. HCV core protein (124,125) or HIV-1 transactivator of transcription (126,127)), presumably with its N-terminal helicase domain, blocking its interactions with other protein partners, such as TRBP or ADAR1 (126,127). In addition, viral proteins may mediate the proteasomal degradation of Dicer, such as HIV-1 protein R (128).

Interactions between Dicer and other proteins can also influence the specificity of its action (129–131). For example, it has been demonstrated that the Dicer C-terminus functions as a 5-lipooxygenase (5LO)-binding domain (129). The association of these two proteins not only enhances the catalytic activity of 5LO but also modifies Dicer processing specificity towards pre-miRNAs, favouring the production of ∼55-nt and ∼10- to 12-nt-long RNA species (129). It is worth noting that years ago, Dicer cDNA clones were isolated from a yeast two-hybrid screen using 5LO as a bait (132). In humans, 5LO is mainly expressed in differentiated inflammatory cells, where it catalyzes the first two steps in the biosynthesis of potent inflammation mediators called leukotrienes (133). The exact nature and impact of Dicer-5LO interactions remain to be determined; nevertheless, the existing evidence provides a link between Dicer and inflammatory processes.

Several reports have shown that Dicer is subject to caspase-dependent degradation during apoptosis in human cells *in vitro* (130,134) and in *C. elegans* (131). The caspase-mediated cleavage of Dicer results in the release of a C-terminal Dicer fragment containing RNase IIIb and dsRBD. This C-terminal Dicer fragment cannot naturally produce functional miRNAs and lacks the majority of domains essential for binding to other proteins and factors. Instead, in *C. elegans*, this C-terminal fragment acts as a deoxyribonuclease to cause breaks in chromosomal DNA (131). Nevertheless, it is not clear whether the human ribonuclease Dicer also participates directly in chromosome fragmentation following caspase cleavage. Interestingly, the caspase-dependent cleavage of human Dicer has been observed during the late stages of HIV-1 infection *in vitro* (130).

**Figure 2.**
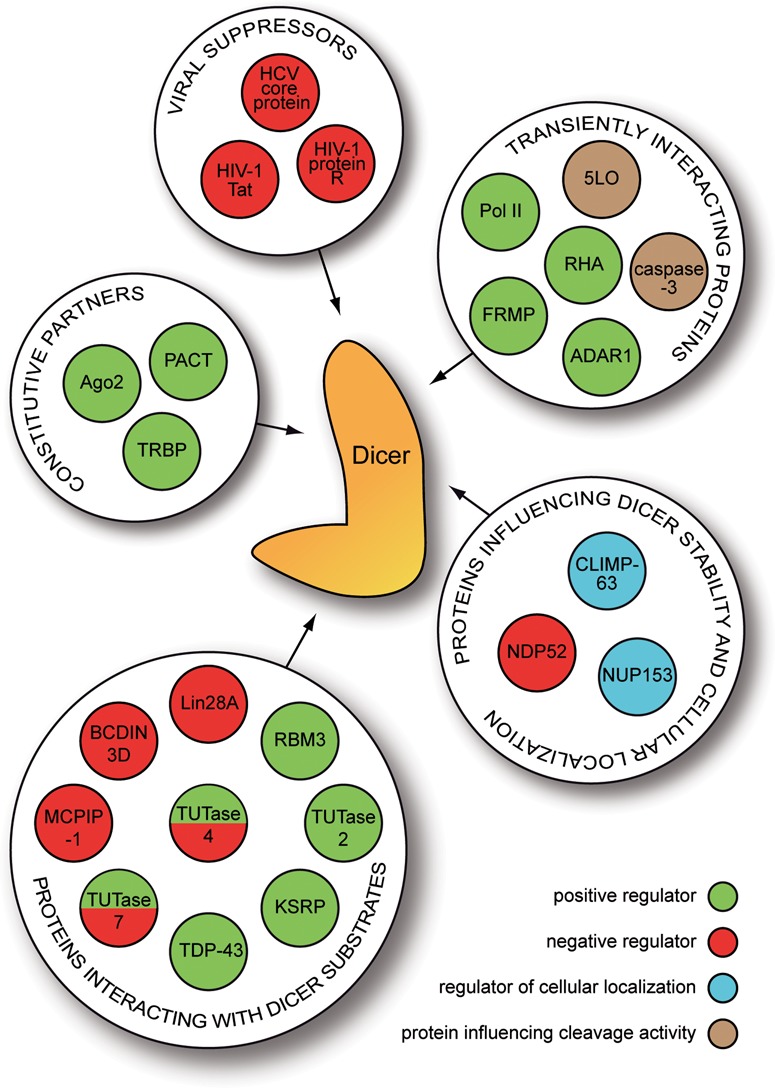
Proteins interacting with human ribonuclease Dicer and its substrates. 5LO—5 lipoxygenase, ADAR1—adenosine deaminase acting on RNA 1, Ago2—Argonaute protein 2, BCDIN3D—BCDIN3 Domain-Containing Protein, CLIMP-63—cytoskeleton-linking membrane protein 63 kDa, FMRP—fragile X mental retardation protein, HIV-1 Tat—HIV-1 transactivator of transcription, KSRP—KH-type splicing regulatory protein, MCPIP-1—monocyte chemoattractant protein-induced protein 1, NDP52—nuclear dot protein 52 kDa, NUP153—nucleoporin 153, PACT—protein activator of interferon-induced protein kinase, Pol II—RNA polymerase II, RBM3—RNA binding motif protein 3, RHA—RNA helicase A, TDP-43—TAR DNA-binding protein-43, TRBP—HIV-1 trans-activation response element RNA-binding protein, TUTase 2/4/7—3′ terminal uridylyl transferase 2/4/7. Summary description of Dicer interacting proteins can be found in Supplementary Table S1.

Obviously, the Dicer–protein interaction network is still being delineated. The current list of involved proteins is summarized in Figure 2 and Supplementary Table S1.

## FACTORS INTERACTING WITH DICER SUBSTRATES

Although the structure of pre-miRNA itself may influence the specificity of precursor cleavage by Dicer (135), an additional regulatory layer of Dicer activity is indicated by the interactions of factors with its substrates. Daley *et al*. have demonstrated that Lin28, a highly conserved RNA-binding protein, selectively blocks the processing of let-7 pre-miRNAs by Dicer through specific binding with these precursors (136,137). Furthermore, Gregory *et* *al*. have shown that a conserved cytosine residue in the loop of pre-let-7g is essential for Lin28 binding (138). In contrast, Kim *et* *al*. have emphasized a role of a tetra-nucleotide sequence motif (GGAG) present in the terminal loop of let-7 miRNA family precursors in their selective binding with Lin28 (139). These authors have demonstrated that Lin28 recruits a terminal uridylyl transferase (TUTase) to let-7 pre-miRNAs. This noncanonical polymerase adds an oligouridine tail to let-7 pre-miRNAs. These uridylated precursors cannot be efficiently processed by Dicer, and they are subsequently targeted for degradation. Lin28-dependent uridylation has also been reported for several other pre-miRNAs that contain the GGAG sequence motif in their apical loops (139,140). A more recent report has indicated that up to three Lin28 molecules assemble in a stepwise manner at the terminal loop region of let-7 miRNA family precursors, thereby efficiently inhibiting their processing by Dicer (141).

A novel mechanism that controls Dicer activity through the Lin28/let-7 axis has been recently described by Varelas *et* *al*. (142). This mechanism involves two major effector proteins of the Hippo kinase signalling pathways, TAZ and YAP (142). The Hippo pathway is an evolutionally conserved signalling cascade that controls organ size and stem cell fate through the regulation of cell proliferation and apoptosis (143,144). The depletion of nuclear TAZ/YAP through the activation of the Hippo pathway results in the reduction of Lin28 and the increased expression of let-7 miRNAs (142). Consequently, let-7 miRNAs can target Dicer mRNA by the mechanism discussed earlier and downregulate its expression, affecting global levels of miRNAs (142).

In contrast with Lin28, a KH-type splicing regulatory protein (KSRP) has been determined to positively influence let-7 miRNA biogenesis (145–147). KSRP is a multi-functional protein that plays roles in the decay (148,149), splicing (150) and localization (151) of certain mRNAs. It has also been reported as a component of Drosha and Dicer complexes in cultured cells (146). It can interact with guanosine-rich motifs located in the apical loops of some miRNA precursors to promote their processing (145,146,152–154), possibly by optimizing the positioning and/or recruitment of miRNA-generating complexes (146).

It has also been demonstrated that the processing of certain pre-miRNAs occurs under the control of TAR DNA-binding protein-43 (TDP-43). TDP-43 interacts with Dicer and promotes the processing of some pre-miRNAs by binding to their terminal loops (155). A recent study has shown that TDP-43 preferentially binds to UG- and pyrimidine-rich sequences (156–158). However, it has also been reported that UG repeats are neither necessary nor sufficient for TDP-43 binding (159) and that TDP-43 cannot bind to UG repeats in the dsRNA region of pre-miR-574. These findings indicate that the RNA secondary structure also plays an important role in target recognition by TDP-43 (155).

The importance of the pre-miRNA structure in the regulation of Dicer activity has been further highlighted in a recent study performed by Wang *et* *al*. (34). These authors have shown that the products of pre-miR-151 editing by the ADAR family protein (varying in their apical loop structures) induce different conformational changes in the Dicer helicase domain upon binding. The characteristics of these changes are correlated with the dicing activity of the enzyme (34).

Another protein targeting the terminal loop of pre-miRNAs is the mammalian immunoregulator MCPIP1 (monocyte chemoattractant protein [MCP]-1-induced protein 1) (160). Miyazono *et* *al*. have demonstrated that MCPIP1 suppresses miRNA biosynthesis by cleaving the apical loops of pre-miRNAs. These authors have proposed that the loops of some miRNA precursors contain regulatory elements that can activate their degradation (160). The broader list of proteins that interact with Dicer substrates is presented in Supplementary Table S2.

Among the elements that affect Dicer activity through interaction with its substrates there is also a group of non-protein factors. For example, it has been reported that the *in vitro* dicing of guanosine-rich short-hairpin RNAs can be inhibited by quadruplex-binding compounds, such as certain phorphyrazines and bis-quinolinium (161). Dicer activity can also be influenced by the binding of RNA molecules other than substrates to this enzyme. Very recently, human transcriptome-wide analysis has identified so-called ‘passive’ Dicer binding sites (162). These sites are preferentially located in coding sequences and 3′-UTRs that adopt stem-loop structures. The latter, however, are different from the structures of typical pre-miRNAs. Dicer has been shown to be capable of binding but not of cutting passive sites. Interactions with Dicer stabilize RNAs carrying passive sites. Passive binding has also been proposed to serve as an anchoring mechanism for the efficient assembly of protein complexes. In addition, passive sites may function as a buffering system to control the catalytic activity of the enzyme by sequestering it from other targets. A similar strategy, based on Dicer sequestering, is utilized by viruses to mislead host defence mechanisms. For example, adenoviruses protect their RNAs by producing high amounts of long self-complementary transcripts that effectively compete for Dicer binding with other endogenous Dicer substrates. As a result, pivotal viral transcripts are not cleaved (163). Likewise, *in vitro* studies conducted by our group have indicated that the activity of human Dicer can be affected by short RNA molecules that are bound to it (164). Detailed studies have revealed that short RNAs can not only act as competitive or allosteric inhibitors of Dicer but can also influence this enzyme by base pairing with its substrates (165). We have found that RNA oligomers that can simultaneously bind both Dicer and its substrates are selective and effective inhibitors of pre-miRNA processing. Furthermore, we have demonstrated that RNAs as short as 12 nt promote the selective inhibition of complementary pre-miRNA cleavage by Dicer (165). The results of several recent studies support our observations that RNA may function as both a substrate and a regulator of miRNA pathway components. For example, Pasquinelli *et* *al*. have identified an interesting auto-regulatory loop that controls let-7 miRNA biogenesis in *C. elegans* (166) involving a protein called ALG-1 (Argonaute-like protein-1) that binds to a specific site at the 3′ end of let-7 primary transcripts (let-7 pri-miRNAs), promoting the processing of these precursors. The interaction between ALG-1 and let-7 pri-miRNA is mediated by mature let-7 miRNA through a conserved complementary site in let-7 pri-miRNA. Therefore, mature miRNAs may target and regulate the processing of their precursor non-coding RNAs. This finding of the auto-regulation of let-7 miRNA biogenesis provides novel insights into the mechanisms controlling miRNA production. Interestingly, Provost *et* *al*. have reported the discovery of 12-nt-long RNA species corresponding with the 5′ regions of miRNAs termed semi-miRNAs (smiRNAs) (167). The data collected by these authors suggest that smiRNAs can compete with miRNAs for binding sites within target UTRs. Thus, smiRNAs may represent a novel class of small non-coding RNAs generated along the miRNA pathway that are capable of regulating the activities of the miRNAs from which they are derived.

The cytoplasm of cells contains RNA molecules of different sizes and types, including a fraction of small regulatory RNAs. Accordingly, short sequence motifs influencing Dicer activity may occur in large functional RNAs, e.g. in mRNAs or in the stable intermediates formed during their degradation. The theory that stable intermediates of RNA degradation can accumulate in the cell and function as signalling molecules or participate in mechanisms that control cellular pathways has been discussed extensively by Figlerowicz *et* *al*. (168). Moreover, it has been demonstrated that products of RNA degradation may be involved in regulatory processes occurring in cells (169,170). Interestingly, we have also identified transcripts whose fragments display substantial similarity to oligomers that bind to human Dicer and affect its activity (164). Consequently, recent findings have indicated that mutual interactions between miRNA precursors and other RNAs may form a very complex regulatory network that controls miRNA biogenesis and subsequent gene expression.

## HUMAN DICER AND CANCER

The human Dicer gene is located within the subtelomeric region 14q32.13 that has been reported to be significantly affected by various mutations (common mutations, epimutations and copy number variations) (171,172). Ample evidence shows that mutations of these types may alter *DICER1* expression and/or resultant protein activity and consequently initiate pathological processes (173–175). Although a number of such mutations have been identified to date, pathomechanisms of Dicer-mutation-mediated diseases are still poorly understood. This fact is well exemplified by neoplastic diseases and has been recently thoroughly discussed by Foulkes, Priest and Duchaine (176). Thus, here we would like to point out only the most important issues.

There is no clear correlation among *DICER1* expression, cancer type and disease progression. For example, significant changes in *DICER1* expression have been detected during different stages of lung adenocarcinoma (177). A transient upregulation in Dicer gene expression has been observed during the early stages of lung adenocarcinoma, whereas it is downregulated during the more advanced stages of this cancer (177). In addition, the reduced expression of *DICER1* may be associated with poor prognosis in some types of lung cancers (178). In contrast, its expression has been shown to be increased in prostate adenocarcinoma cancer (179) and Burkitt's lymphoma (180). The levels of Dicer mRNA/protein accumulation in select cancers are shown in Supplementary Table S3.

There are also many controversies whether *DICER1* acts as a tumour suppressor or an oncogene (181). In humans, both constitutional (Supplementary Figure S1) and somatic (Supplementary Table S4) *DICER1* mutations have been identified The former are inherited (germline mutations) and present in every cell and the latter are not inherited and occur in some, usually small, fraction of cells or even in a single cell. Early studies involving mouse models suggested that *DICER1* functions as a haploinsufficient tumour suppressor (182,183). The haploinsufficiency mechanism proposed for *DICER1* postulates that the constitutional mutation inactivating one allele of *DICER1* (a heterozygous germline mutation), is initiatory and predisposes a cell to neoplastic disease; however, some other events are also required to induce tumorigenesis (184). Such constitutional, loss-of-function mutations have been found throughout *DICER1* (176,184) and they have been proposed to lead to the so called *DICER1* syndrome associated with various, usually early childhood cancers (184). Recently, several reports that support another model of Dicer-mutation-mediated tumourigenesis, so called ‘two-hit’ tumor suppressor model, have been published (173,175). This model is based on a classic Knundon's hypothesis assuming that two hits are needed to inactivate a tumor suppressor gene (185). The first hit inactivates one allele and occurs either in somatic cell (sporadic cancer) or in germline cell (hereditary cancer). The second hit, affecting a remaining wild-type allele, is always a somatic mutation. Interestingly, in case of *DICER1*, most of the second hit mutations have been found in the RNase IIIb domain (173,175,186–192). These mutations usually inactivate RNase IIIb only and specifically change Dicer activity. As mentioned earlier, Dicer lacking the functional RNase IIIb domain fails to cleave miRNAs located in the 5′-arms of pre-miRNA hairpins. As a result, miRNAs located in the 3′-arms of pre-miRNA hairpins are mainly produced (62,174).

The recent findings are consistent with the observations that certain families of miRNAs are indispensable for tumor cell development (11,193,194) that is why the inactivation of both *DICER1* alleles suppresses cancer (183). Thus, one can hypothesize that a selective pressure operating in cancer cells acts against complete loss of *DICER1* and supports the appearance of specific mutations that are not deleterious for the encoded protein. These mutations rather modify Dicer activity toward restricting the formation of tumor suppressor miRNAs and/or promoting the production of oncogenic miRNAs. Considering all these data, one can further speculate that *DICER1* can function both as a tumor suppressor and an oncogene. Finally, it should also be noted that there are many cancers for which neither germline nor somatic *DICER1* mutations have a major etiological role.

## CONCLUSIONS AND PERSPECTIVES

**Figure 3.**
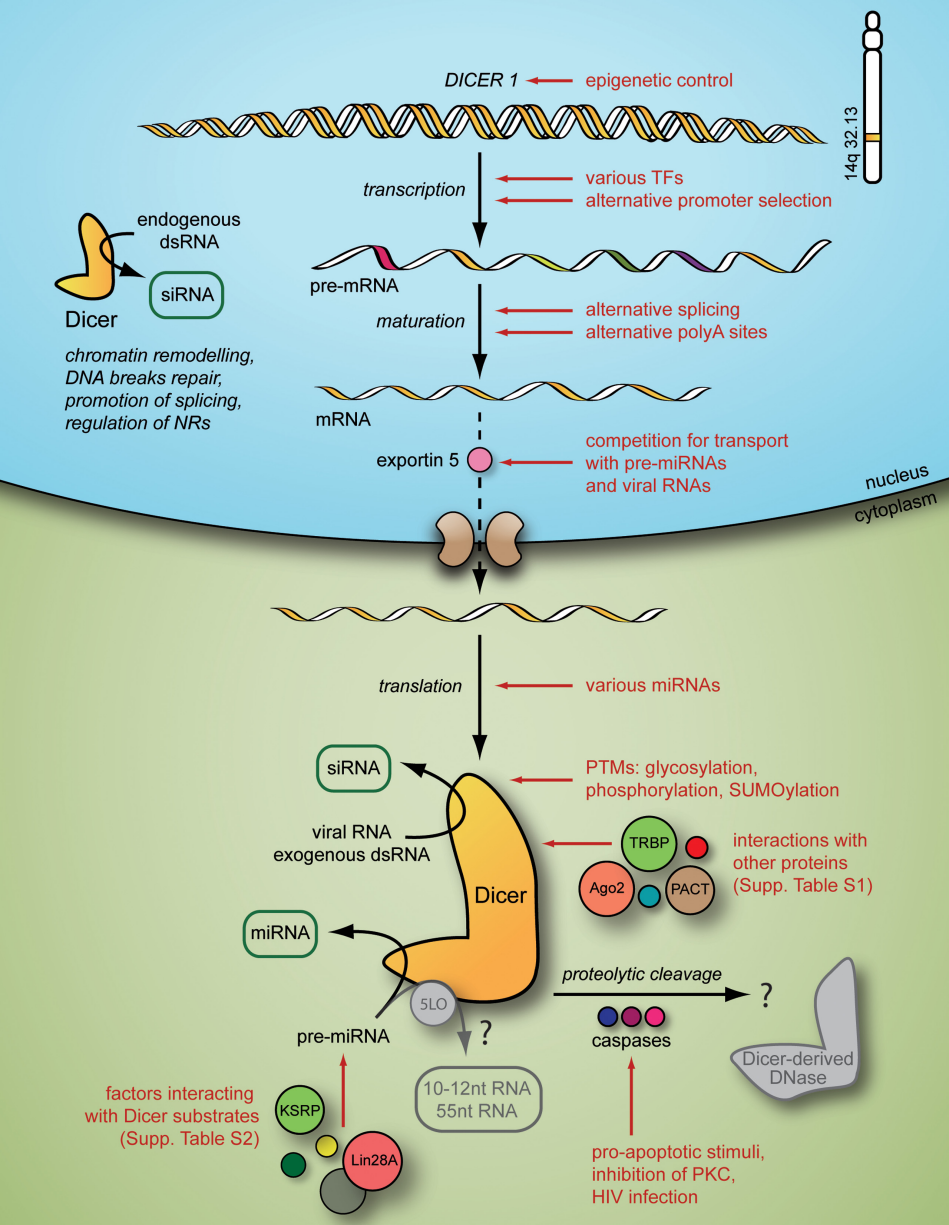
The widespread regulation of Dicer biosynthesis and function in human cells. 5LO—5 lipoxygenase, Ago2—Argonaute protein 2, KSRP—KH-type splicing regulatory protein, NRs—nuclear receptors, PACT—protein activator of interferon-induced protein kinase, PKC—protein kinase C, PTMs—post-translational modifications, TFs—transcription factors and TRBP—HIV-1 trans-activation response element RNA-binding protein. The hypothetical functions of Dicer, which have not been proven for the human enzyme, are presented in translucent grey and are indicated with a question mark.

The precise regulation of Dicer activity is critical for the proper functioning of all eukaryotic organisms. As discussed in this paper and summarized in Figure 3, Dicer abundance, activity and specificity can be regulated by various types of factors and at multiple levels. However, despite intensive studies, many basic questions regarding the mechanisms regulating the activity of this enzyme remain unanswered.

In the past few years, several reports have indicated the existence of interplay between Dicer and factors involved in various important cellular pathways. One can assume that the term ‘Dicer interaction network’ may refer exclusively to the Dicer protein, e.g. the contribution of the Dicer protein to chromatin structure remodelling, apoptosis or inflammation, as reviewed above. However, the involvement of Dicer mRNA in this interplay has also been documented. As discussed earlier, competition for nuclear exportin binding among Dicer mRNAs, pre-miRNAs and viral RNAs may affect Dicer protein levels (69). In addition, viral RNAs may influence the Dicer protein directly by its sequestration, which also prevents the maturation of host pre-miRNAs to functional miRNAs (163), as described in this review. Considering other conditions and details of cell functioning, the issue of the Dicer interaction network becomes much more complicated. Certainly, solving one problem leads to further questions. Thus, to obtain a more comprehensive image of the Dicer interaction network, systems biology, a new and increasingly popular biological discipline, must be considered. Nevertheless, there is no doubt that the elucidation of any aspects of the mechanisms underlying Dicer activity will represent valuable contributions to the understanding of numerous phenomena, including developmental timing, growth, differentiation, apoptosis and viral infections.

## SUPPLEMENTARY DATA

Supplementary Data are available at NAR Online.

SUPPLEMENTARY DATA

## References

[1] Ambros V. (2003). MicroRNA pathways in flies and worms: growth, death, fat, stress, and timing. Cell.

[2] Carthew R.W. (2006). Gene regulation by microRNAs. Curr. Opin. Genet. Dev..

[3] Kim V.N. (2005). MicroRNA biogenesis: coordinated cropping and dicing. Nat. Rev. Mol. Cell Biol..

[4] Friedman R.C., Farh K.K., Burge C.B., Bartel D.P. (2009). Most mammalian mRNAs are conserved targets of microRNAs. Genome Res..

[5] Olejniczak M., Galka P., Krzyzosiak W.J. (2009). Sequence-non-specific effects of RNA interference triggers and microRNA regulators. Nucleic Acids Res..

[6] Berkhout B., Haasnoot J. (2006). The interplay between virus infection and the cellular RNA interference machinery. FEBS Lett..

[7] Haasnoot J., Berkhout B. (2006). Handb Exp Pharmacol.

[8] Kurzynska-Kokorniak A., Jackowiak P., Figlerowicz M. (2009). Human- and virus-encoded microRNAs as potential targets of antiviral therapy. Mini Rev. Med. Chem..

[9] Jackowiak P., Figlerowicz M., Kurzynska-Kokorniak A. (2011). Mechanisms involved in the development of chronic hepatitis C as potential targets of antiviral therapy. Curr. Pharm. Biotechnol..

[10] Calin G.A., Croce C.M. (2006). MicroRNA signatures in human cancers. Nat. Rev. Cancer.

[11] Esquela-Kerscher A., Slack F.J. (2006). Oncomirs—microRNAs with a role in cancer. Nat. Rev. Cancer.

[12] Hebert S.S., De Strooper B. (2009). Alterations of the microRNA network cause neurodegenerative disease. Trends Neurosci..

[13] Tili E., Michaille J.J., Costinean S., Croce C.M. (2008). MicroRNAs, the immune system and rheumatic disease. Nat. Clin. Pract. Rheumatol..

[14] Bernstein E., Caudy A.A., Hammond S.M., Hannon G.J. (2001). Role for a bidentate ribonuclease in the initiation step of RNA interference. Nature.

[15] Macrae I.J., Li F., Zhou K., Cande W.Z., Doudna J.A. (2006). Structure of Dicer and mechanistic implications for RNAi. Cold Spring Harb. Symp. Quant. Biol..

[16] Macrae I.J., Zhou K., Li F., Repic A., Brooks A.N., Cande W.Z., Adams P.D., Doudna J.A. (2006). Structural basis for double-stranded RNA processing by Dicer. Science.

[17] Zhang H., Kolb F.A., Brondani V., Billy E., Filipowicz W. (2002). Human Dicer preferentially cleaves dsRNAs at their termini without a requirement for ATP. EMBO J..

[18] Zhang H., Kolb F.A., Jaskiewicz L., Westhof E., Filipowicz W. (2004). Single processing center models for human Dicer and bacterial RNase III. Cell.

[19] Lau P.W., Guiley K.Z., De N., Potter C.S., Carragher B., MacRae I.J. (2012). The molecular architecture of human Dicer. Nat. Struct. Mol. Biol..

[20] Lau P.W., Potter C.S., Carragher B., MacRae I.J. (2009). Structure of the human Dicer-TRBP complex by electron microscopy. Structure.

[21] Taylor D.W., Ma E., Shigematsu H., Cianfrocco M.A., Noland C.L., Nagayama K., Nogales E., Doudna J.A., Wang H.W. (2013). Substrate-specific structural rearrangements of human Dicer. Nat. Struct. Mol. Biol..

[22] Wang H.W., Noland C., Siridechadilok B., Taylor D.W., Ma E., Felderer K., Doudna J.A., Nogales E. (2009). Structural insights into RNA processing by the human RISC-loading complex. Nat. Struct. Mol. Biol..

[23] Tian Y., Simanshu D.K., Ma J.B., Park J.E., Heo I., Kim V.N., Patel D.J. (2014). A phosphate-binding pocket within the platform-PAZ-connector helix cassette of human Dicer. Mol. Cell.

[24] Takeshita D., Zenno S., Lee W.C., Nagata K., Saigo K., Tanokura M. (2007). Homodimeric structure and double-stranded RNA cleavage activity of the C-terminal RNase III domain of human dicer. J. Mol. Biol..

[25] Dlakic M. (2006). DUF283 domain of Dicer proteins has a double-stranded RNA-binding fold. Bioinformatics.

[26] Lingel A., Simon B., Izaurralde E., Sattler M. (2003). Structure and nucleic-acid binding of the Drosophila Argonaute 2 PAZ domain. Nature.

[27] Ma J.B., Ye K., Patel D.J. (2004). Structural basis for overhang-specific small interfering RNA recognition by the PAZ domain. Nature.

[28] Song J.J., Liu J., Tolia N.H., Schneiderman J., Smith S.K., Martienssen R.A., Hannon G.J., Joshua-Tor L. (2003). The crystal structure of the Argonaute2 PAZ domain reveals an RNA binding motif in RNAi effector complexes. Nat. Struct. Biol..

[29] Yan K.S., Yan S., Farooq A., Han A., Zeng L., Zhou M.M. (2003). Structure and conserved RNA binding of the PAZ domain. Nature.

[30] Park J.E., Heo I., Tian Y., Simanshu D.K., Chang H., Jee D., Patel D.J., Kim V.N. (2011). Dicer recognizes the 5′ end of RNA for efficient and accurate processing. Nature.

[31] Ma E., Zhou K., Kidwell M.A., Doudna J.A. (2012). Coordinated activities of human dicer domains in regulatory RNA processing. J. Mol. Biol..

[32] Gu S., Jin L., Zhang Y., Huang Y., Zhang F., Valdmanis P.N., Kay M.A. (2012). The loop position of shRNAs and pre-miRNAs is critical for the accuracy of dicer processing in vivo. Cell.

[33] Tsutsumi A., Kawamata T., Izumi N., Seitz H., Tomari Y. (2011). Recognition of the pre-miRNA structure by Drosophila Dicer-1. Nat. Struct. Mol. Biol..

[34] Liu Z., Wang J., Li G., Wang H.W. (2014). Structure of precursor microRNA's terminal loop regulates human Dicer's dicing activity by switching DExH/D domain. Protein Cell.

[35] Lee Y., Hur I., Park S.Y., Kim Y.K., Suh M.R., Kim V.N. (2006). The role of PACT in the RNA silencing pathway. EMBO J..

[36] Ye X., Paroo Z., Liu Q. (2007). Functional anatomy of the Drosophila microRNA-generating enzyme. J. Biol. Chem..

[37] Ma E., MacRae I.J., Kirsch J.F., Doudna J.A. (2008). Autoinhibition of human dicer by its internal helicase domain. J. Mol. Biol..

[38] Cerutti H., Casas-Mollano J.A. (2006). On the origin and functions of RNA-mediated silencing: from protists to man. Curr. Genet..

[39] Mukherjee K., Campos H., Kolaczkowski B. (2012). Evolution of animal and plant dicers: early parallel duplications and recurrent adaptation of antiviral RNA binding in plants. Mol. Biol. Evol..

[40] Drinnenberg I.A., Fink G.R., Bartel D.P. (2011). Compatibility with killer explains the rise of RNAi-deficient fungi. Science.

[41] Nicolas F.E., Torres-Martinez S., Ruiz-Vazquez R.M. (2013). Loss and retention of RNA interference in fungi and parasites. PLoS Pathog..

[42] Lee Y.S., Nakahara K., Pham J.W., Kim K., He Z., Sontheimer E.J., Carthew R.W. (2004). Distinct roles for Drosophila Dicer-1 and Dicer-2 in the siRNA/miRNA silencing pathways. Cell.

[43] Czech B., Malone C.D., Zhou R., Stark A., Schlingeheyde C., Dus M., Perrimon N., Kellis M., Wohlschlegel J.A., Sachidanandam R. (2008). An endogenous small interfering RNA pathway in Drosophila. Nature.

[44] Galiana-Arnoux D., Dostert C., Schneemann A., Hoffmann J.A., Imler J.L. (2006). Essential function in vivo for Dicer-2 in host defense against RNA viruses in drosophila. Nat. Immunol..

[45] Singh S., Bevan S.C., Patil K., Newton D.C., Marsden P.A. (2005). Extensive variation in the 5′-UTR of Dicer mRNAs influences translational efficiency. Biochem. Biophys. Res. Commun..

[46] Irvin-Wilson C.V., Chaudhuri G. (2005). Alternative initiation and splicing in dicer gene expression in human breast cells. Breast Cancer Res..

[47] Jafarnejad S.M., Ardekani G.S., Ghaffari M., Martinka M., Li G. (2013). Sox4-mediated Dicer expression is critical for suppression of melanoma cell invasion. Oncogene.

[48] Aaboe M., Birkenkamp-Demtroder K., Wiuf C., Sorensen F.B., Thykjaer T., Sauter G., Jensen K.M., Dyrskjot L., Orntoft T. (2006). SOX4 expression in bladder carcinoma: clinical aspects and in vitro functional characterization. Cancer Res..

[49] Lee C.J., Appleby V.J., Orme A.T., Chan W.I., Scotting P.J. (2002). Differential expression of SOX4 and SOX11 in medulloblastoma. J. Neurooncol..

[50] Rhodes D.R., Yu J., Shanker K., Deshpande N., Varambally R., Ghosh D., Barrette T., Pandey A., Chinnaiyan A.M. (2004). Large-scale meta-analysis of cancer microarray data identifies common transcriptional profiles of neoplastic transformation and progression. Proc. Natl. Acad. Sci. U.S.A..

[51] Liu P., Ramachandran S., Ali Seyed M., Scharer C.D., Laycock N., Dalton W.B., Williams H., Karanam S., Datta M.W., Jaye D.L. (2006). Sex-determining region Y box 4 is a transforming oncogene in human prostate cancer cells. Cancer Res..

[52] Chiosea S., Jelezcova E., Chandran U., Acquafondata M., McHale T., Sobol R.W., Dhir R. (2006). Up-regulation of dicer, a component of the MicroRNA machinery, in prostate adenocarcinoma. Am. J. Pathol..

[53] Levy C., Khaled M., Robinson K.C., Veguilla R.A., Chen P.H., Yokoyama S., Makino E., Lu J., Larue L., Beermann F. (2010). Lineage-specific transcriptional regulation of DICER by MITF in melanocytes. Cell.

[54] Boominathan L. (2010). The guardians of the genome (p53, TA-p73, and TA-p63) are regulators of tumor suppressor miRNAs network. Cancer Metastasis. Rev..

[55] Wiesen J.L., Tomasi T.B. (2009). Dicer is regulated by cellular stresses and interferons. Mol. Immunol..

[56] Saayman S., Ackley A., Turner A.M., Famiglietti M., Bosque A., Clemson M., Planelles V., Morris K.V. (2014). An HIV-encoded antisense long noncoding RNA epigenetically regulates viral transcription. Mol. Ther..

[57] Froberg J.E., Yang L., Lee J.T. (2013). Guided by RNAs: X-inactivation as a model for lncRNA function. J. Mol. Biol..

[58] Matsuda S., Ichigotani Y., Okuda T., Irimura T., Nakatsugawa S., Hamaguchi M. (2000). Molecular cloning and characterization of a novel human gene (HERNA) which encodes a putative RNA-helicase. Biochim. Biophys. Acta.

[59] Grelier G., Voirin N., Ay A.S., Cox D.G., Chabaud S., Treilleux I., Leon-Goddard S., Rimokh R., Mikaelian I., Venoux C. (2009). Prognostic value of Dicer expression in human breast cancers and association with the mesenchymal phenotype. Br. J. Cancer.

[60] Hinkal G.W., Grelier G., Puisieux A., Moyret-Lalle C. (2011). Complexity in the regulation of Dicer expression: Dicer variant proteins are differentially expressed in epithelial and mesenchymal breast cancer cells and decreased during EMT. Br. J. Cancer.

[61] Potenza N., Papa U., Scaruffi P., Mosca N., Tonini G.P., Russo A. (2010). A novel splice variant of the human dicer gene is expressed in neuroblastoma cells. FEBS Lett..

[62] Gurtan A.M., Lu V., Bhutkar A., Sharp P.A. (2012). In vivo structure-function analysis of human Dicer reveals directional processing of precursor miRNAs. RNA.

[63] Rakheja D., Chen K.S., Liu Y., Shukla A.A., Schmid V., Chang T.C., Khokhar S., Wickiser J.E., Karandikar N.J., Malter J.S. (2014). Somatic mutations in DROSHA and DICER1 impair microRNA biogenesis through distinct mechanisms in Wilms tumours. Nat. Commun..

[64] Martello G., Rosato A., Ferrari F., Manfrin A., Cordenonsi M., Dupont S., Enzo E., Guzzardo V., Rondina M., Spruce T. (2010). A MicroRNA targeting dicer for metastasis control. Cell.

[65] Feinberg-Gorenshtein G., Guedj A., Shichrur K., Jeison M., Luria D., Kodman Y., Ash S., Feinmesser M., Edry L., Shomron N. (2013). MiR-192 directly binds and regulates Dicer1 expression in neuroblastoma. PLoS One.

[66] Forman J.J., Legesse-Miller A., Coller H.A. (2008). A search for conserved sequences in coding regions reveals that the let-7 microRNA targets Dicer within its coding sequence. Proc. Natl. Acad. Sci. U.S.A..

[67] Mayr C., Bartel D.P. (2009). Widespread shortening of 3′ UTRs by alternative cleavage and polyadenylation activates oncogenes in cancer cells. Cell.

[68] Hamaya Y., Kuriyama S., Takai T., Yoshida K., Yamada T., Sugimoto M., Osawa S., Sugimoto K., Miyajima H., Kanaoka S. (2012). A distinct expression pattern of the long 3′-untranslated region dicer mRNA and its implications for posttranscriptional regulation in colorectal cancer. Clin. Transl. Gastroenterol..

[69] Bennasser Y., Chable-Bessia C., Triboulet R., Gibbings D., Gwizdek C., Dargemont C., Kremer E.J., Voinnet O., Benkirane M. (2011). Competition for XPO5 binding between Dicer mRNA, pre-miRNA and viral RNA regulates human Dicer levels. Nat. Struct. Mol. Biol..

[70] Rigbolt K.T., Prokhorova T.A., Akimov V., Henningsen J., Johansen P.T., Kratchmarova I., Kassem M., Mann M., Olsen J.V., Blagoev B. (2011). System-wide temporal characterization of the proteome and phosphoproteome of human embryonic stem cell differentiation. Sci. Signal.

[71] Gross T.J., Powers L.S., Boudreau R.L., Brink B., Reisetter A., Goel K., Gerke A.K., Hassan I.H., Monick M.M. (2014). A microRNA processing defect in smokers’ macrophages is linked to SUMOylation of the endonuclease DICER. J. Biol. Chem..

[72] Paroo Z., Ye X., Chen S., Liu Q. (2009). Phosphorylation of the human microRNA-generating complex mediates MAPK/Erk signaling. Cell.

[73] Drake M., Furuta T., Suen K.M., Gonzalez G., Liu B., Kalia A., Ladbury J.E., Fire A.Z., Skeath J.B., Arur S. (2014). A requirement for ERK-dependent Dicer phosphorylation in coordinating oocyte-to-embryo transition in C. elegans. Dev. Cell.

[74] Pepin G., Perron M.P., Provost P. (2012). Regulation of human Dicer by the resident ER membrane protein CLIMP-63. Nucleic Acids Res..

[75] Chendrimada T.P., Gregory R.I., Kumaraswamy E., Norman J., Cooch N., Nishikura K., Shiekhattar R. (2005). TRBP recruits the Dicer complex to Ago2 for microRNA processing and gene silencing. Nature.

[76] Haase A.D., Jaskiewicz L., Zhang H., Laine S., Sack R., Gatignol A., Filipowicz W. (2005). TRBP, a regulator of cellular PKR and HIV-1 virus expression, interacts with Dicer and functions in RNA silencing. EMBO Rep..

[77] Lee H.Y., Zhou K., Smith A.M., Noland C.L., Doudna J.A. (2013). Differential roles of human Dicer-binding proteins TRBP and PACT in small RNA processing. Nucleic Acids Res..

[78] Takahashi T., Miyakawa T., Zenno S., Nishi K., Tanokura M., Ui-Tei K. (2013). Distinguishable in vitro binding mode of monomeric TRBP and dimeric PACT with siRNA. PLoS One.

[79] Fukunaga R., Han B.W., Hung J.H., Xu J., Weng Z., Zamore P.D. (2012). Dicer partner proteins tune the length of mature miRNAs in flies and mammals. Cell.

[80] Chakravarthy S., Sternberg S.H., Kellenberger C.A., Doudna J.A. (2010). Substrate-specific kinetics of Dicer-catalyzed RNA processing. J. Mol. Biol..

[81] Lee H.Y., Doudna J.A. (2012). TRBP alters human precursor microRNA processing in vitro. RNA.

[82] Kok K.H., Ng M.H., Ching Y.P., Jin D.Y. (2007). Human TRBP and PACT directly interact with each other and associate with dicer to facilitate the production of small interfering RNA. J. Biol. Chem..

[83] Koscianska E., Starega-Roslan J., Krzyzosiak W.J. (2011). The role of Dicer protein partners in the processing of microRNA precursors. PLoS One.

[84] MacRae I.J., Ma E., Zhou M., Robinson C.V., Doudna J.A. (2008). In vitro reconstitution of the human RISC-loading complex. Proc. Natl. Acad. Sci. U.S.A..

[85] Mourelatos Z., Dostie J., Paushkin S., Sharma A., Charroux B., Abel L., Rappsilber J., Mann M., Dreyfuss G. (2002). miRNPs: a novel class of ribonucleoproteins containing numerous microRNAs. Genes Dev..

[86] Hutvagner G., Zamore P.D. (2002). A microRNA in a multiple-turnover RNAi enzyme complex. Science.

[87] Gregory R.I., Chendrimada T.P., Cooch N., Shiekhattar R. (2005). Human RISC couples microRNA biogenesis and posttranscriptional gene silencing. Cell.

[88] Filipowicz W. (2005). RNAi: the nuts and bolts of the RISC machine. Cell.

[89] Pratt A.J., MacRae I.J. (2009). The RNA-induced silencing complex: a versatile gene-silencing machine. J. Biol. Chem..

[90] Maniataki E., Mourelatos Z. (2005). A human, ATP-independent, RISC assembly machine fueled by pre-miRNA. Genes Dev..

[91] Noland C.L., Ma E., Doudna J.A. (2011). siRNA repositioning for guide strand selection by human Dicer complexes. Mol. Cell.

[92] Kanellopoulou C., Muljo S.A., Kung A.L., Ganesan S., Drapkin R., Jenuwein T., Livingston D.M., Rajewsky K. (2005). Dicer-deficient mouse embryonic stem cells are defective in differentiation and centromeric silencing. Genes Dev..

[93] Martinez J., Patkaniowska A., Urlaub H., Luhrmann R., Tuschl T. (2002). Single-stranded antisense siRNAs guide target RNA cleavage in RNAi. Cell.

[94] Ohrt T., Mutze J., Staroske W., Weinmann L., Hock J., Crell K., Meister G., Schwille P. (2008). Fluorescence correlation spectroscopy and fluorescence cross-correlation spectroscopy reveal the cytoplasmic origination of loaded nuclear RISC in vivo in human cells. Nucleic Acids Res..

[95] Roberts T.C. (2014). The microRNA biology of the mammalian nucleus. Mol. Ther. Nucleic Acids.

[96] Ota H., Sakurai M., Gupta R., Valente L., Wulff B.E., Ariyoshi K., Iizasa H., Davuluri R.V., Nishikura K. (2013). ADAR1 forms a complex with Dicer to promote microRNA processing and RNA-induced gene silencing. Cell.

[97] Kawahara Y., Zinshteyn B., Chendrimada T.P., Shiekhattar R., Nishikura K. (2007). RNA editing of the microRNA-151 precursor blocks cleavage by the Dicer-TRBP complex. EMBO Rep..

[98] Yang W., Chendrimada T.P., Wang Q., Higuchi M., Seeburg P.H., Shiekhattar R., Nishikura K. (2006). Modulation of microRNA processing and expression through RNA editing by ADAR deaminases. Nat. Struct. Mol. Biol..

[99] Daniels S.M., Melendez-Pena C.E., Scarborough R.J., Daher A., Christensen H.S., El Far M., Purcell D.F., Laine S., Gatignol A. (2009). Characterization of the TRBP domain required for dicer interaction and function in RNA interference. BMC Mol. Biol..

[100] Provost P., Dishart D., Doucet J., Frendewey D., Samuelsson B., Radmark O. (2002). Ribonuclease activity and RNA binding of recombinant human Dicer. EMBO J..

[101] Tahbaz N., Kolb F.A., Zhang H., Jaronczyk K., Filipowicz W., Hobman T.C. (2004). Characterization of the interactions between mammalian PAZ PIWI domain proteins and Dicer. EMBO Rep..

[102] Sinkkonen L., Hugenschmidt T., Filipowicz W., Svoboda P. (2010). Dicer is associated with ribosomal DNA chromatin in mammalian cells. PLoS One.

[103] Peng J.C., Karpen G.H. (2007). H3K9 methylation and RNA interference regulate nucleolar organization and repeated DNA stability. Nat. Cell. Biol..

[104] Cam H.P., Sugiyama T., Chen E.S., Chen X., FitzGerald P.C., Grewal S.I. (2005). Comprehensive analysis of heterochromatin- and RNAi-mediated epigenetic control of the fission yeast genome. Nat. Genet..

[105] Gullerova M., Proudfoot N.J. (2012). Convergent transcription induces transcriptional gene silencing in fission yeast and mammalian cells. Nat. Struct. Mol. Biol..

[106] Fukagawa T., Nogami M., Yoshikawa M., Ikeno M., Okazaki T., Takami Y., Nakayama T., Oshimura M. (2004). Dicer is essential for formation of the heterochromatin structure in vertebrate cells. Nat. Cell. Biol..

[107] Hall I.M., Shankaranarayana G.D., Noma K., Ayoub N., Cohen A., Grewal S.I. (2002). Establishment and maintenance of a heterochromatin domain. Science.

[108] Verdel A., Jia S., Gerber S., Sugiyama T., Gygi S., Grewal S.I., Moazed D. (2004). RNAi-mediated targeting of heterochromatin by the RITS complex. Science.

[109] Noma K., Sugiyama T., Cam H., Verdel A., Zofall M., Jia S., Moazed D., Grewal S.I. (2004). RITS acts in cis to promote RNA interference-mediated transcriptional and post-transcriptional silencing. Nat. Genet..

[110] White E., Schlackow M., Kamieniarz-Gdula K., Proudfoot N.J., Gullerova M. (2014). Human nuclear Dicer restricts the deleterious accumulation of endogenous double-stranded RNA. Nat. Struct. Mol. Biol..

[111] Volpe T.A., Kidner C., Hall I.M., Teng G., Grewal S.I., Martienssen R.A. (2002). Regulation of heterochromatic silencing and histone H3 lysine-9 methylation by RNAi. Science.

[112] Ameyar-Zazoua M., Rachez C., Souidi M., Robin P., Fritsch L., Young R., Morozova N., Fenouil R., Descostes N., Andrau J.C. (2012). Argonaute proteins couple chromatin silencing to alternative splicing. Nat. Struct. Mol. Biol..

[113] Haussecker D., Proudfoot N.J. (2005). Dicer-dependent turnover of intergenic transcripts from the human beta-globin gene cluster. Mol. Cell. Biol..

[114] Tang K.F., Wang Y., Wang P., Chen M., Chen Y., Hu H.D., Hu P., Wang B., Yang W., Ren H. (2007). Upregulation of PHLDA2 in Dicer knockdown HEK293 cells. Biochim. Biophys. Acta.

[115] Allo M., Buggiano V., Fededa J.P., Petrillo E., Schor I., de la Mata M., Agirre E., Plass M., Eyras E., Elela S.A. (2009). Control of alternative splicing through siRNA-mediated transcriptional gene silencing. Nat. Struct. Mol. Biol..

[116] Cramer P., Caceres J.F., Cazalla D., Kadener S., Muro A.F., Baralle F.E., Kornblihtt A.R. (1999). Coupling of transcription with alternative splicing: RNA pol II promoters modulate SF2/ASF and 9G8 effects on an exonic splicing enhancer. Mol. Cell.

[117] Brodsky A.S., Meyer C.A., Swinburne I.A., Hall G., Keenan B.J., Liu X.S., Fox E.A., Silver P.A. (2005). Genomic mapping of RNA polymerase II reveals sites of co-transcriptional regulation in human cells. Genome Biol..

[118] Francia S., Michelini F., Saxena A., Tang D., de Hoon M., Anelli V., Mione M., Carninci P., d'Adda di Fagagna F. (2012). Site-specific DICER and DROSHA RNA products control the DNA-damage response. Nature.

[119] Wei W., Ba Z., Gao M., Wu Y., Ma Y., Amiard S., White C.I., Rendtlew Danielsen J.M., Yang Y.G., Qi Y. (2012). A role for small RNAs in DNA double-strand break repair. Cell.

[120] Michalik K.M., Bottcher R., Forstemann K. (2012). A small RNA response at DNA ends in Drosophila. Nucleic Acids Res..

[121] Redfern A.D., Colley S.M., Beveridge D.J., Ikeda N., Epis M.R., Li X., Foulds C.E., Stuart L.M., Barker A., Russell V.J. (2013). RNA-induced silencing complex (RISC) Proteins PACT, TRBP, and Dicer are SRA binding nuclear receptor coregulators. Proc. Natl. Acad. Sci. U.S.A..

[122] Murchison E.P., Stein P., Xuan Z., Pan H., Zhang M.Q., Schultz R.M., Hannon G.J. (2007). Critical roles for Dicer in the female germline. Genes Dev..

[123] Tang F., Kaneda M., O'Carroll D., Hajkova P., Barton S.C., Sun Y.A., Lee C., Tarakhovsky A., Lao K., Surani M.A. (2007). Maternal microRNAs are essential for mouse zygotic development. Genes Dev..

[124] Chen W., Zhang Z., Chen J., Zhang J., Wu Y., Huang Y., Cai X., Huang A. (2008). HCV core protein interacts with Dicer to antagonize RNA silencing. Virus Res..

[125] Wang Y., Kato N., Jazag A., Dharel N., Otsuka M., Taniguchi H., Kawabe T., Omata M. (2006). Hepatitis C virus core protein is a potent inhibitor of RNA silencing-based antiviral response. Gastroenterology.

[126] Bennasser Y., Jeang K.T. (2006). HIV-1 Tat interaction with Dicer: requirement for RNA. Retrovirology.

[127] Bennasser Y., Yeung M.L., Jeang K.T. (2006). HIV-1 TAR RNA subverts RNA interference in transfected cells through sequestration of TAR RNA-binding protein, TRBP. J. Biol. Chem..

[128] Casey Klockow L., Sharifi H.J., Wen X., Flagg M., Furuya A.K., Nekorchuk M., de Noronha C.M. (2013). The HIV-1 protein Vpr targets the endoribonuclease Dicer for proteasomal degradation to boost macrophage infection. Virology.

[129] Dincbas-Renqvist V., Pepin G., Rakonjac M., Plante I., Ouellet D.L., Hermansson A., Goulet I., Doucet J., Samuelsson B., Radmark O. (2009). Human Dicer C-terminus functions as a 5-lipoxygenase binding domain. Biochim. Biophys. Acta.

[130] Matskevich A.A., Moelling K. (2008). Stimuli-dependent cleavage of Dicer during apoptosis. Biochem. J..

[131] Nakagawa A., Shi Y., Kage-Nakadai E., Mitani S., Xue D. (2010). Caspase-dependent conversion of Dicer ribonuclease into a death-promoting deoxyribonuclease. Science.

[132] Provost P., Samuelsson B., Radmark O. (1999). Interaction of 5-lipoxygenase with cellular proteins. Proc. Natl. Acad. Sci. U.S.A..

[133] Radmark O., Werz O., Steinhilber D., Samuelsson B. (2007). 5-Lipoxygenase: regulation of expression and enzyme activity. Trends Biochem. Sci..

[134] Ghodgaonkar M.M., Shah R.G., Kandan-Kulangara F., Affar E.B., Qi H.H., Wiemer E., Shah G.M. (2009). Abrogation of DNA vector-based RNAi during apoptosis in mammalian cells due to caspase-mediated cleavage and inactivation of Dicer-1. Cell Death Differ..

[135] Starega-Roslan J., Koscianska E., Kozlowski P., Krzyzosiak W.J. (2011). The role of the precursor structure in the biogenesis of microRNA. Cell. Mol. Life Sci..

[136] Viswanathan S.R., Daley G.Q. (2010). Lin28: a microRNA regulator with a macro role. Cell.

[137] Viswanathan S.R., Daley G.Q., Gregory R.I. (2008). Selective blockade of microRNA processing by Lin28. Science.

[138] Piskounova E., Viswanathan S.R., Janas M., LaPierre R.J., Daley G.Q., Sliz P., Gregory R.I. (2008). Determinants of microRNA processing inhibition by the developmentally regulated RNA-binding protein Lin28. J. Biol. Chem..

[139] Heo I., Joo C., Kim Y.K., Ha M., Yoon M.J., Cho J., Yeom K.H., Han J., Kim V.N. (2009). TUT4 in concert with Lin28 suppresses microRNA biogenesis through pre-microRNA uridylation. Cell.

[140] Newman M.A., Mani V., Hammond S.M. (2011). Deep sequencing of microRNA precursors reveals extensive 3′ end modification. RNA.

[141] Desjardins A., Bouvette J., Legault P. (2014). Stepwise assembly of multiple Lin28 proteins on the terminal loop of let-7 miRNA precursors. Nucleic Acids Res..

[142] Chaulk S.G., Lattanzi V.J., Hiemer S.E., Fahlman R.P., Varelas X. (2013). The Hippo pathway effectors TAZ/YAP regulate dicer expression and microRNA biogenesis through Let-7. J. Biol. Chem..

[143] Pan D. (2010). The hippo signaling pathway in development and cancer. Dev. Cell.

[144] Zhao B., Tumaneng K., Guan K.L. (2011). The Hippo pathway in organ size control, tissue regeneration and stem cell self-renewal. Nat. Cell Biol..

[145] Nicastro G., Garcia-Mayoral M.F., Hollingworth D., Kelly G., Martin S.R., Briata P., Gherzi R., Ramos A. (2012). Noncanonical G recognition mediates KSRP regulation of let-7 biogenesis. Nat. Struct. Mol. Biol..

[146] Trabucchi M., Briata P., Garcia-Mayoral M., Haase A.D., Filipowicz W., Ramos A., Gherzi R., Rosenfeld M.G. (2009). The RNA-binding protein KSRP promotes the biogenesis of a subset of microRNAs. Nature.

[147] Michlewski G., Caceres J.F. (2010). Antagonistic role of hnRNP A1 and KSRP in the regulation of let-7a biogenesis. Nat. Struct. Mol. Biol..

[148] Gherzi R., Lee K.Y., Briata P., Wegmuller D., Moroni C., Karin M., Chen C.Y. (2004). A KH domain RNA binding protein, KSRP, promotes ARE-directed mRNA turnover by recruiting the degradation machinery. Mol. Cell.

[149] Chou C.F., Mulky A., Maitra S., Lin W.J., Gherzi R., Kappes J., Chen C.Y. (2006). Tethering KSRP, a decay-promoting AU-rich element-binding protein, to mRNAs elicits mRNA decay. Mol. Cell. Biol..

[150] Min H., Turck C.W., Nikolic J.M., Black D.L. (1997). A new regulatory protein, KSRP, mediates exon inclusion through an intronic splicing enhancer. Genes Dev..

[151] Gu W., Pan F., Zhang H., Bassell G.J., Singer R.H. (2002). A predominantly nuclear protein affecting cytoplasmic localization of beta-actin mRNA in fibroblasts and neurons. J. Cell Biol..

[152] Briata P., Lin W.J., Giovarelli M., Pasero M., Chou C.F., Trabucchi M., Rosenfeld M.G., Chen C.Y., Gherzi R. (2012). PI3K/AKT signaling determines a dynamic switch between distinct KSRP functions favoring skeletal myogenesis. Cell Death Differ..

[153] Ruggiero T., Trabucchi M., De Santa F., Zupo S., Harfe B.D., McManus M.T., Rosenfeld M.G., Briata P., Gherzi R. (2009). LPS induces KH-type splicing regulatory protein-dependent processing of microRNA-155 precursors in macrophages. FASEB J..

[154] Zhang X., Wan G., Berger F.G., He X., Lu X. (2011). The ATM kinase induces microRNA biogenesis in the DNA damage response. Mol. Cell.

[155] Kawahara Y., Mieda-Sato A. (2012). TDP-43 promotes microRNA biogenesis as a component of the Drosha and Dicer complexes. Proc. Natl. Acad. Sci. U.S.A..

[156] Xiao S., Sanelli T., Dib S., Sheps D., Findlater J., Bilbao J., Keith J., Zinman L., Rogaeva E., Robertson J. (2011). RNA targets of TDP-43 identified by UV-CLIP are deregulated in ALS. Mol. Cell. Neurosci..

[157] Tollervey J.R., Curk T., Rogelj B., Briese M., Cereda M., Kayikci M., Konig J., Hortobagyi T., Nishimura A.L., Zupunski V. (2011). Characterizing the RNA targets and position-dependent splicing regulation by TDP-43. Nat. Neurosci..

[158] Sephton C.F., Cenik C., Kucukural A., Dammer E.B., Cenik B., Han Y., Dewey C.M., Roth F.P., Herz J., Peng J. (2011). Identification of neuronal RNA targets of TDP-43-containing ribonucleoprotein complexes. J. Biol. Chem..

[159] Polymenidou M., Lagier-Tourenne C., Hutt K.R., Huelga S.C., Moran J., Liang T.Y., Ling S.C., Sun E., Wancewicz E., Mazur C. (2011). Long pre-mRNA depletion and RNA missplicing contribute to neuronal vulnerability from loss of TDP-43. Nat. Neurosci..

[160] Suzuki H.I., Arase M., Matsuyama H., Choi Y.L., Ueno T., Mano H., Sugimoto K., Miyazono K. (2011). MCPIP1 ribonuclease antagonizes dicer and terminates microRNA biogenesis through precursor microRNA degradation. Mol. Cell.

[161] Henn A., Joachimi A., Goncalves D.P., Monchaud D., Teulade-Fichou M.P., Sanders J.K., Hartig J.S. (2008). Inhibition of dicing of guanosine-rich shRNAs by quadruplex-binding compounds. Chembiochem.

[162] Rybak-Wolf A., Jens M., Murakawa Y., Herzog M., Landthaler M., Rajewsky N. (2014). A variety of Dicer substrates in human and C. elegans. Cell.

[163] Andersson M.G., Haasnoot P.C., Xu N., Berenjian S., Berkhout B., Akusjarvi G. (2005). Suppression of RNA interference by adenovirus virus-associated RNA. J. Virol..

[164] Tyczewska A., Kurzynska-Kokorniak A., Koralewska N., Szopa A., Kietrys A.M., Wrzesinski J., Twardowski T., Figlerowicz M. (2011). Selection of RNA oligonucleotides that can modulate human dicer activity in vitro. Nucleic Acid Ther..

[165] Kurzynska-Kokorniak A., Koralewska N., Tyczewska A., Twardowski T., Figlerowicz M. (2013). A new short oligonucleotide-based strategy for the precursor-specific regulation of microRNA processing by dicer. PLoS One.

[166] Zisoulis D.G., Kai Z.S., Chang R.K., Pasquinelli A.E. (2012). Autoregulation of microRNA biogenesis by let-7 and Argonaute. Nature.

[167] Plante I., Ple H., Landry P., Gunaratne P.H., Provost P. (2012). Modulation of microRNA Activity by Semi-microRNAs. Front. Genet..

[168] Jackowiak P., Nowacka M., Strozycki P.M., Figlerowicz M. (2011). RNA degradome–its biogenesis and functions. Nucleic Acids Res..

[169] Mercer T.R., Dinger M.E., Bracken C.P., Kolle G., Szubert J.M., Korbie D.J., Askarian-Amiri M.E., Gardiner B.B., Goodall G.J., Grimmond S.M. (2010). Regulated post-transcriptional RNA cleavage diversifies the eukaryotic transcriptome. Genome Res..

[170] Nowacka M., Strozycki P.M., Jackowiak P., Hojka-Osinska A., Szymanski M., Figlerowicz M. (2013). Identification of stable, high copy number, medium-sized RNA degradation intermediates that accumulate in plants under non-stress conditions. Plant Mol. Biol..

[171] Kagami M., Sekita Y., Nishimura G., Irie M., Kato F., Okada M., Yamamori S., Kishimoto H., Nakayama M., Tanaka Y. (2008). Deletions and epimutations affecting the human 14q32.2 imprinted region in individuals with paternal and maternal upd(14)-like phenotypes. Nat. Genet..

[172] Zhang L., Huang J., Yang N., Greshock J., Megraw M.S., Giannakakis A., Liang S., Naylor T.L., Barchetti A., Ward M.R. (2006). microRNAs exhibit high frequency genomic alterations in human cancer. Proc. Natl. Acad. Sci. U.S.A..

[173] Heravi-Moussavi A., Anglesio M.S., Cheng S.W., Senz J., Yang W., Prentice L., Fejes A.P., Chow C., Tone A., Kalloger S.E. (2012). Recurrent somatic DICER1 mutations in nonepithelial ovarian cancers. N. Engl. J. Med..

[174] Anglesio M.S., Wang Y., Yang W., Senz J., Wan A., Heravi-Moussavi A., Salamanca C., Maines-Bandiera S., Huntsman D.G., Morin G.B. (2013). Cancer-associated somatic DICER1 hotspot mutations cause defective miRNA processing and reverse-strand expression bias to predominantly mature 3p strands through loss of 5p strand cleavage. J. Pathol..

[175] Wu M.K., Sabbaghian N., Xu B., Addidou-Kalucki S., Bernard C., Zou D., Reeve A.E., Eccles M.R., Cole C., Choong C.S. (2013). Biallelic DICER1 mutations occur in Wilms tumours. J. Pathol..

[176] Foulkes W.D., Priest J.R., Duchaine T.F. (2014). DICER1: mutations, microRNAs and mechanisms. Nat. Rev. Cancer.

[177] Chiosea S., Jelezcova E., Chandran U., Luo J., Mantha G., Sobol R.W., Dacic S. (2007). Overexpression of Dicer in precursor lesions of lung adenocarcinoma. Cancer Res..

[178] Karube Y., Tanaka H., Osada H., Tomida S., Tatematsu Y., Yanagisawa K., Yatabe Y., Takamizawa J., Miyoshi S., Mitsudomi T. (2005). Reduced expression of Dicer associated with poor prognosis in lung cancer patients. Cancer Sci..

[179] Ambs S., Prueitt R.L., Yi M., Hudson R.S., Howe T.M., Petrocca F., Wallace T.A., Liu C.G., Volinia S., Calin G.A. (2008). Genomic profiling of microRNA and messenger RNA reveals deregulated microRNA expression in prostate cancer. Cancer Res..

[180] Kaul D., Sikand K. (2004). Defective RNA-mediated c-myc gene silencing pathway in Burkitt's lymphoma. Biochem. Biophys. Res. Commun..

[181] Klein S., Lee H., Ghahremani S., Kempert P., Ischander M., Teitell M.A., Nelson S.F., Martinez-Agosto J.A. (2014). Expanding the phenotype of mutations in DICER1: mosaic missense mutations in the RNase IIIb domain of DICER1 cause GLOW syndrome. J. Med. Genet..

[182] Kumar M.S., Pester R.E., Chen C.Y., Lane K., Chin C., Lu J., Kirsch D.G., Golub T.R., Jacks T. (2009). Dicer1 functions as a haploinsufficient tumor suppressor. Genes Dev..

[183] Lambertz I., Nittner D., Mestdagh P., Denecker G., Vandesompele J., Dyer M.A., Marine J.C. (2010). Monoallelic but not biallelic loss of Dicer1 promotes tumorigenesis in vivo. Cell Death Differ..

[184] Slade I., Bacchelli C., Davies H., Murray A., Abbaszadeh F., Hanks S., Barfoot R., Burke A., Chisholm J., Hewitt M. (2011). DICER1 syndrome: clarifying the diagnosis, clinical features and management implications of a pleiotropic tumour predisposition syndrome. J. Med. Genet..

[185] Knudson A.G. Jr (1971). Mutation and cancer: statistical study of retinoblastoma. Proc. Natl. Acad. Sci. U.S.A..

[186] de Kock L., Sabbaghian N., Soglio D.B., Guillerman R.P., Park B.K., Chami R., Deal C.L., Priest J.R., Foulkes W.D. (2014). Exploring the association Between DICER1 mutations and differentiated thyroid carcinoma. J. Clin. Endocrinol. Metab..

[187] de Kock L., Plourde F., Carter M.T., Hamel N., Srivastava A., Meyn M.S., Arseneau J., Bouron-Dal Soglio D., Foulkes W.D. (2013). Germ-line and somatic DICER1 mutations in a pleuropulmonary blastoma. Pediatr. Blood Cancer.

[188] de Kock L., Sabbaghian N., Plourde F., Srivastava A., Weber E., Bouron-Dal Soglio D., Hamel N., Choi J.H., Park S.H., Deal C.L. (2014). Pituitary blastoma: a pathognomonic feature of germ-line DICER1 mutations. Acta Neuropathol..

[189] Seki M., Yoshida K., Shiraishi Y., Shimamura T., Sato Y., Nishimura R., Okuno Y., Chiba K., Tanaka H., Kato K. (2014). Biallelic DICER1 mutations in sporadic pleuropulmonary blastoma. Cancer Res..

[190] Murray M.J., Bailey S., Raby K.L., Saini H.K., de Kock L., Burke G.A., Foulkes W.D., Enright A.J., Coleman N., Tischkowitz M. (2014). Serum levels of mature microRNAs in DICER1-mutated pleuropulmonary blastoma. Oncogenesis.

[191] Tomiak E., de Kock L., Grynspan D., Ramphal R., Foulkes W.D. (2014). DICER1 mutations in an adolescent with cervical embryonal rhabdomyosarcoma (cERMS). Pediatr. Blood Cancer.

[192] Sahakitrungruang T., Srichomthong C., Pornkunwilai S., Amornfa J., Shuangshoti S., Kulawonganunchai S., Suphapeetiporn K., Shotelersuk V. (2014). Germline and somatic DICER1 mutations in a pituitary ibBlastoma causing infantile-onset Cushing's disease. J. Clin. Endocrinol. Metab..

[193] Cho W.C. (2007). OncomiRs: the discovery and progress of microRNAs in cancers. Mol. Cancer.

[194] Krutovskikh V.A., Herceg Z. (2010). Oncogenic microRNAs (OncomiRs) as a new class of cancer biomarkers. Bioessays.

