# Supplementary material for: The many faces of Dicer: the complexity of the mechanisms regulating Dicer gene expression and enzyme activities
Source: Nucleic Acids Res. 2015 Apr 16;43(9):4365–80. doi: 10.1093/nar/gkv328 (PMC4482082; doi:10.1093/nar/gkv328)
Supplement: SUPPLEMENTARY DATA [file supp_43_9_4365__index.html]

The many faces of Dicer: the complexity of the mechanisms regulating Dicer gene expression and enzyme activities — SUPPLEMENTARY DATA 

# The many faces of Dicer: the complexity of the mechanisms regulating Dicer gene expression and enzyme activities

## SUPPLEMENTARY DATA

**Files in this Data Supplement:**

- SUPPLEMENTARY DATA
