# Supplementary material for: The many faces of Dicer: the complexity of the mechanisms regulating Dicer gene expression and enzyme activities
Source: Nucleic Acids Res. 2015 Apr 16;43(9):4365–80. doi: 10.1093/nar/gkv328 (PMC4482082; doi:10.1093/nar/gkv328)
Supplement: SUPPLEMENTARY DATA [file supp_gkv328_nar-03037-survey-d-2014-File005.pdf]

The many faces of Dicer: the complexity of the mechanisms regulating Dicer gene expression and enzyme activities

Anna Kurzynska-Kokorniak[1], Natalia Koralewska[1], Maria Pokornowska[1], Anna Urbanowicz[1], Aleksander Tworak[1], Agnieszka Mickiewicz[1] and Marek Figlerowicz[1,2,*]

[1] Institute of Bioorganic Chemistry, Polish Academy of Sciences, Poznan, 61-704, Poland
[2] Institute of Computing Science, Poznan University of Technology, Poznan, 60-965, Poland

* To whom correspondence should be addressed. Tel: +48 618 528 503 ext. 103; fax: +48 618 520 532; email: marekf@ibch.poznan.pl

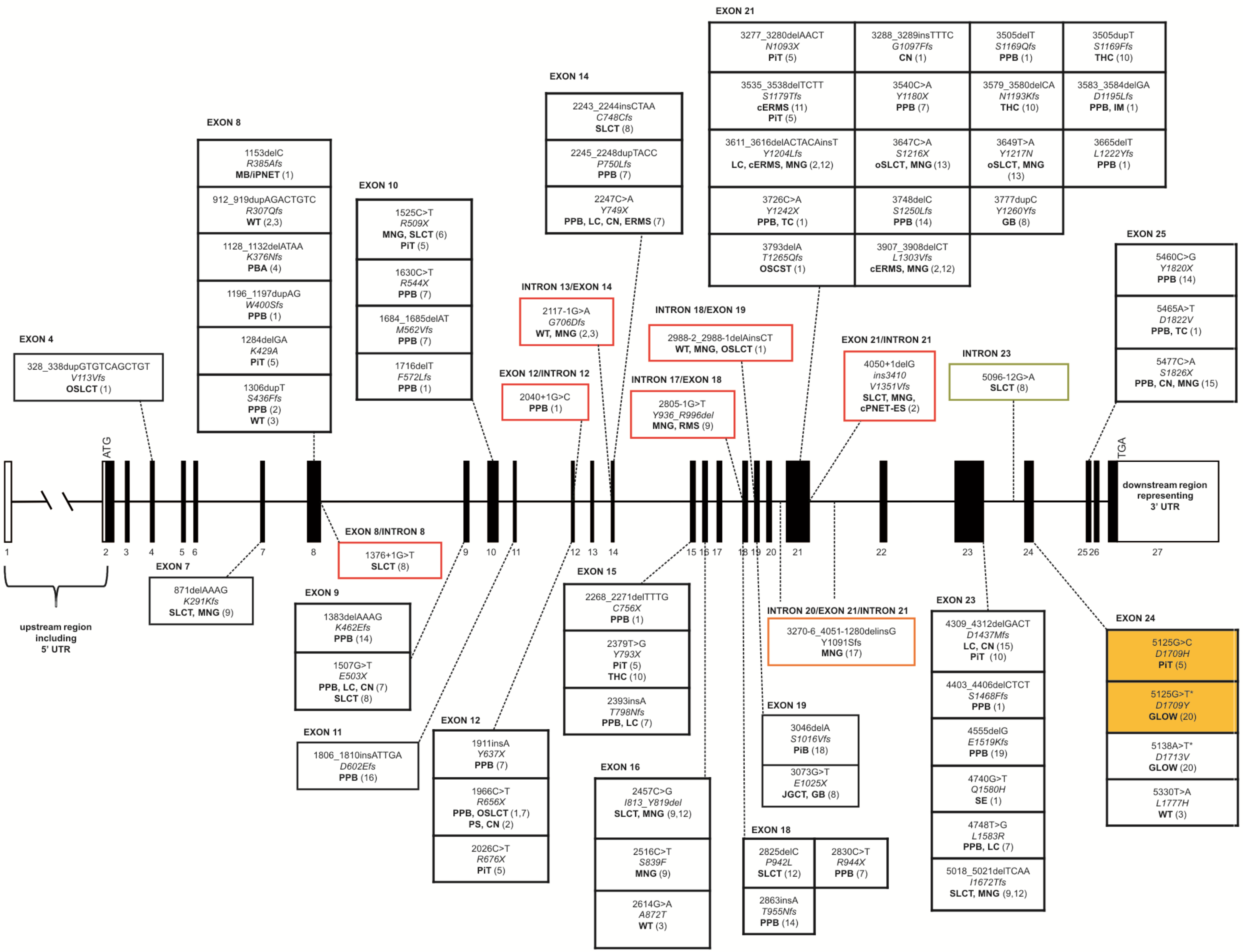

EXON 4
328_338dupGTGTCAGCTGT V113Vfs OSLCT (1)
EXON 8
1153delC R385Afs MB/iPNET (1)
912_919dupAGACTGTC R307Qfs WT (2,3)
1128_1132delATAA K376Nfs PBA (4)
1196_1197dupAG W400Sfs PPB (1)
1284delGA K429A PiT (5)
1306dupT S436Ffs PPB (2) WT (3)
EXON 10
1525C>T R509X MNG, SLCT (6) PiT (5)
1630C>T R544X PPB (7)
1684_1685delAT M562Vfs PPB (7)
1716delT F572Lfs PPB (1)
EXON 14
2243_2244insCTAA C748Cfs SLCT (8)
2245_2248dupTACC P750Lfs PPB (7)
2247C>A Y749X PPB, LC, CN, ERMS (7)
INTRON 13/EXON 14
2117-1G>A G706Dfs WT, MNG (2,3)
EXON 12/INTRON 12
2040+1G>C PPB (1)
INTRON 18/EXON 19
2988-2_2988-1delAinsCT WT, MNG, OSLCT (1)
INTRON 17/EXON 18
2805-1G>T Y936_R996del MNG, RMS (9)
EXON 21
3277_3280delAACT N1093X PiT (5)
3288_3289insTTTC G1097Ffs CN (1)
3505delT S1169Qfs PPB (1)
3505dupT S1169Ffs THC (10)
3535_3538delTCTT S1179Tfs cERMS (11) PiT (5)
3540C>A Y1180X PPB (7)
3579_3580delCA N1193Kfs THC (10)
3583_3584delGA D1195Lfs PPB, IM (1)
3611_3616delACTACAinsT Y1204Lfs LC, cERMS, MNG (2,12)
3647C>A S1216X oSLCT, MNG (13)
3649T>A Y1217N oSLCT, MNG (13)
3665delT L1222Yfs PPB (1)
3726C>A Y1242X PPB, TC (1)
3748delC S1250Lfs PPB (14)
3777dupC Y1260Yfs GB (8)
3793delA T1265Qfs OSCST (1)
3907_3908delCT L1303Vfs cERMS, MNG (2,12)
EXON 21/INTRON 21
4050+1delG ins3410 V1351Vfs SLCT, MNG, cPNET-ES (2)
INTRON 23
5096-12G>A SLCT (8)
EXON 25
5460C>G Y1820X PPB (14)
5465A>T D1822V PPB, TC (1)
5477C>A S1826X PPB, CN, MNG (15)
ATG
TGA
1 2 3 4 5 6 7 8 9 10 11 12 13 14 15 16 17 18 19 20 21 22 23 24 25 26 27
downstream region representing 3' UTR
upstream region including 5' UTR
EXON 7
871delAAAG K291Kfs SLCT, MNG (9)
EXON 8/INTRON 8
1376+1G>T SLCT (8)
EXON 9
1383delAAAG K462Efs PPB (14)
1507G>T E503X PPB, LC, CN (7) SLCT (8)
EXON 11
1806_1810insATTGA D602Efs PPB (16)
EXON 12
1911insA Y637X PPB (7)
1966C>T R656X PPB, OSLCT (1,7) PS, CN (2)
2026C>T R676X PiT (5)
EXON 15
2268_2271delTTTG C756X PPB (1)
2379T>G Y793X PiT (5) THC (10)
2393insA T798Nfs PPB, LC (7)
EXON 16
2457C>G I813_Y819del SLCT, MNG (9,12)
2516C>T S839F MNG (9)
2614G>A A872T WT (3)
INTRON 20/EXON 21/INTRON 21
3270-6_4051-1280delinsG Y1091Sfs MNG (17)
EXON 19
3046delA S1016Vfs PiB (18)
3073G>T E1025X JGCT, GB (8)
EXON 18
2825delC P942L SLCT (12)
2830C>T R944X PPB (7)
2863insA T955Nfs PPB (14)
EXON 23
4309_4312delGACT D1437Mfs LC, CN (15) PiT (10)
4403_4406delCTCT S1468Ffs PPB (1)
4555delG E1519Kfs PPB (19)
4740G>T Q1580H SE (1)
4748T>G L1583R PPB, LC (7)
5018_5021delTCAA I1672Tfs SLCT, MNG (9,12)
EXON 24
5125G>C D1709H PiT (5)
5125G>T* D1709Y GLOW (20)
5138A>T* D1713V GLOW (20)
5330T>A L1777H WT (3)

Supplementary Figure S1. Heritable mutations in *DICER1* gene

The genomic (NG_016311) and transcript (NM_177438.2) reference sequences were used to notate human Dicer cDNA and protein (*in italics*) sequences. The phenotype of patients bearing the mutations studied is described **in bold**. Mutations framed in black boxes constitute non-synonymous or frame-shift exonic mutations. Mutations framed in red boxes constitute mutations located in the intron-exon splice sites. The mutation framed in the orange box is a whole exon deletion. The mutation framed in the green box is an intronic mutation with unknown influence on transcript or protein sequences. Mutations colored in yellow affect the amino acid residue responsible for metal binding in the catalytic center of RNase IIIb domain.

Abbreviations: cERMS – cervical embryonal rhabdomyosarcoma, CN – cystic nephroma, cPNET-ES cervicalprimitive neuroepithelial tumor-Ewing sarcoma, del – deletion, dup – duplication, ERMS – embryonal rhabdomyosarcoma, fs – frame-shift, GB – gynandroblastoma, GLOW – tumors associated with GLOW syndrome, IM – intraocular medulloepithelioma, ins – insertion, JGCT – juvenile granulosa cell tumor, LC – lung cysts, MB/iPNET – medulloblastoma/infratentorial primitive neuroectodermal tumor, MNG – multinodular goiter, OSCST – ovarian sex cord stromal tumor, oSLCT – ovarian Sertoli-Leydig cell tumor, PBA – pineoblastoma, PiT – pituitary blastoma, PPB – pleuropulmonary blastoma, PS – pulmonary sequestration, RMS – rhabdomyosarcoma, SE – seminoma, SLCT – Sertoli-Leydig cell tumor, TC – thyroid cysts, THC – thyroid carcinoma, WT – Wilms tumor

REFERENCES (SUPP. FIGURE 1)

1. Slade, I., Bacchelli, C., Davies, H., Murray, A., Abbaszadeh, F., Hanks, S., Barfoot, R., Burke, A., Chisholm, J., Hewitt, M. *et al.* (2011) DICER1 syndrome: clarifying the diagnosis, clinical features and management implications of a pleiotropic tumour predisposition syndrome. *J Med Genet*, **48**, 273-278.
2. Foulkes, W.D., Bahubeshi, A., Hamel, N., Pasini, B., Asioli, S., Baynam, G., Choong, C.S., Charles, A., Frieder, R.P., Dishop, M.K. *et al.* (2011) Extending the phenotypes associated with DICER1 mutations. *Hum Mutat*, **32**, 1381-1384.
3. Wu, M.K., Sabbaghian, N., Xu, B., Addidou-Kalucki, S., Bernard, C., Zou, D., Reeve, A.E., Eccles, M.R., Cole, C., Choong, C.S. *et al.* (2013) Biallelic DICER1 mutations occur in Wilms tumours. *J Pathol*, **230**, 154-164.
4. Sabbaghian, N., Hamel, N., Srivastava, A., Albrecht, S., Priest, J.R. and Foulkes, W.D. (2012) Germline DICER1 mutation and associated loss of heterozygosity in a pineoblastoma. *J Med Genet*, **49**, 417-419.
5. de Kock, L., Sabbaghian, N., Plourde, F., Srivastava, A., Weber, E., Bouron-Dal Soglio, D., Hamel, N., Choi, J.H., Park, S.H., Deal, C.L. *et al.* (2014) Pituitary blastoma: a pathognomonic feature of germ-line DICER1 mutations. *Acta Neuropathol*, **128**, 111-122.
6. Darrat, I., Bedoyan, J.K., Chen, M., Schuette, J.L. and Lesperance, M.M. (2013) Novel DICER1 mutation as cause of multinodular goiter in children. *Head Neck*, **35**, E369-371.
7. Hill, D.A., Ivanovich, J., Priest, J.R., Gurnett, C.A., Dehner, L.P., Desruisseau, D., Jarzembowski, J.A., Wikenheiser-Brokamp, K.A., Suarez, B.K., Whelan, A.J. *et al.* (2009) DICER1 mutations in familial pleuropulmonary blastoma. *Science*, **325**, 965.
8. Schultz, K.A., Pacheco, M.C., Yang, J., Williams, G.M., Messinger, Y., Hill, D.A., Dehner, L.P. and Priest, J.R. (2011) Ovarian sex cord-stromal tumors, pleuropulmonary blastoma and DICER1 mutations: a report from the International Pleuropulmonary Blastoma Registry. *Gynecol Oncol*, **122**, 246-250.

9. Rio Frio, T., Bahubeshi, A., Kanellopoulou, C., Hamel, N., Niedziela, M., Sabbaghian, N., Pouchet, C., Gilbert, L., O'Brien, P.K., Serfas, K. *et al.* (2011) DICER1 mutations in familial multinodular goiter with and without ovarian Sertoli-Leydig cell tumors. *JAMA*, **305**, 68-77.
10. de Kock, L., Sabbaghian, N., Soglio, D.B., Guillerman, R.P., Park, B.K., Chami, R., Deal, C.L., Priest, J.R. and Foulkes, W.D. (2014) Exploring the association Between DICER1 mutations and differentiated thyroid carcinoma. *J Clin Endocrinol Metab*, **99**, E1072-1077.
11. Tomiak, E., de Kock, L., Grynspan, D., Ramphal, R. and Foulkes, W.D. (2014) DICER1 mutations in an adolescent with cervical embryonal rhabdomyosarcoma (cERMS). *Pediatr Blood Cancer*, **61**, 568-569.
12. Heravi-Moussavi, A., Anglesio, M.S., Cheng, S.W., Senz, J., Yang, W., Prentice, L., Fejes, A.P., Chow, C., Tone, A., Kalloger, S.E. *et al.* (2012) Recurrent somatic DICER1 mutations in nonepithelial ovarian cancers. *N Engl J Med*, **366**, 234-242.
13. Rossing, M., Gerdes, A.M., Juul, A., Rechnitzer, C., Rudnicki, M., Nielsen, F.C. and Vo Hansen, T. (2014) A novel DICER1 mutation identified in a female with ovarian Sertoli-Leydig cell tumor and multinodular goiter: a case report. *J Med Case Rep*, **8**, 112.
14. Seki, M., Yoshida, K., Shiraishi, Y., Shimamura, T., Sato, Y., Nishimura, R., Okuno, Y., Chiba, K., Tanaka, H., Kato, K. *et al.* (2014) Biallelic DICER1 mutations in sporadic pleuropulmonary blastoma. *Cancer Res*, **74**, 2742-2749.
15. Bahubeshi, A., Bal, N., Rio Frio, T., Hamel, N., Pouchet, C., Yilmaz, A., Bouron-Dal Soglio, D., Williams, G.M., Tischkowitz, M., Priest, J.R. *et al.* (2010) Germline DICER1 mutations and familial cystic nephroma. *J Med Genet*, **47**, 863-866.
16. Murray, M.J., Bailey, S., Raby, K.L., Saini, H.K., de Kock, L., Burke, G.A., Foulkes, W.D., Enright, A.J., Coleman, N. and Tischkowitz, M. (2014) Serum levels of mature microRNAs in DICER1-mutated pleuropulmonary blastoma. *Oncogenesis*, **3**, e87.
17. Sabbaghian, N., Srivastava, A., Hamel, N., Plourde, F., Gajtko-Metera, M., Niedziela, M. and Foulkes, W.D. (2013) Germ-line deletion in DICER1 revealed by a novel MLPA assay using synthetic oligonucleotides. *Eur J Hum Genet*, **22**, 564-567.
18. Sahakitrungruang, T., Srichomthong, C., Pornkunwilai, S., Amornfa, J., Shuangshoti, S., Kulawonganunchai, S., Suphapeetiporn, K. and Shotelersuk, V. (2014) Germline and Somatic DICER1 Mutations in a Pituitary Blastoma Causing Infantile-Onset Cushing's Disease. *J Clin Endocrinol Metab*, **99**, E1487-1492.
19. de Kock, L., Plourde, F., Carter, M.T., Hamel, N., Srivastava, A., Meyn, M.S., Arseneau, J., Bouron-Dal Soglio, D. and Foulkes, W.D. (2013) Germ-line and somatic DICER1 mutations in a pleuropulmonary blastoma. *Pediatr Blood Cancer*, **60**, 2091-2092.
20. Klein, S., Lee, H., Ghahremani, S., Kempert, P., Ischander, M., Teitell, M.A., Nelson, S.F. and Martinez-Agosto, J.A. (2014) Expanding the phenotype of mutations in DICER1: mosaic missense mutations in the RNase IIIb domain of DICER1 cause GLOW syndrome. *J Med Genet*, **51**, 294-302.

Supplementary Table S1. The Dicer – protein interaction network

CATEGORY	PROTEIN	CELLULAR ROLE	NATURE OF THE INTERACTION	RELEVANCE TO DICER	REF.
CONSTITUTIVE PARTNERS OF DICER	Ago2 (argonaute protein 2)	A central effector protein of RNAi and an essential member of RISC; cleaves mRNA under guidance of associated miRNAs.	Direct interaction, involving their PIWI and RNase III domains, assisted by Hsp90.	Takes over RNA products from Dicer.	(1,2)
	PACT (protein activator of the interferon-induced PKR)	A major role in the antiviral activity of interferon as an activator of the interferon-induced PKR; protein involved in RNAi as a Dicer cofactor and a component of human RISC.	Direct interaction, involving the third dsRNA-binding domain (dsRBD) of PACT and the N-terminal region of Dicer.	Strengthens Dicer preference towards pre-miRNA, rather than siRNA precursors; influences miRNA production by changing the cleavage site choice by Dicer; contributes to RISC assembly.	(3-5)
	TRBP (TAR RNA-binding protein)	An integral part of RISC, first identified as a cellular protein that facilitates the replication of human immunodeficiency virus by inhibiting the interferon-induced protein kinase R (PKR) and by increasing translation of viral mRNA.	Direct interaction, involving TRBP Medipal domain (298–366 aa) and Dicer domain located between the ATPase and the helicase motifs (267–431 aa).	Recruits dsRNA substrates to Dicer, and remains associated with RNA to affect its structure and relative positioning on Dicer's active site (4-5-fold increase in activity of Dicer upon binding TRBP); contributes to substrate, and product length determination (isomiRs production); stabilizes Dicer and contributes to RISC formation.	(1,3,6-11)
TRANSIENTLY INTERACTING PROTEINS	ADAR1 (adenosine deaminase acting on RNA 1)	Nuclear deaminase, catalyzes A-to-I editing of dsRNA.	Direct interaction, involving DUF238 and DEAD-box RNA helicase of Dicer.	Increases the maximum rate of pre-miRNA cleavage by Dicer; facilitates loading of miRNA onto the RISC.	(12)
	Caspase-3	Protease, plays a central role in the execution phase of cell apoptosis; interacts with other caspases.	Direct interaction resulting in proteolytic cleavage of Dicer.	Cleaves Dicer; truncated Dicer (C-terminal fragment) acts as a DNase and functions in the propagation of apoptosis.	(13-15)

CATEGORY	PROTEIN	CELLULAR ROLE	NATURE OF THE INTERACTION	RELEVANCE TO DICER	REF.
TRANSIENTLY INTERACTING PROTEINS	RHA (RNA helicase A)	ATP-dependent RNA helicase that unwinds RNA-RNA and RNA-DNA duplexes in 3'->5' direction.	Direct interaction with Dicer, but also TRBP, Ago2 and RNA.	RISC-associated protein, promotes the interaction of guide-strand siRNA and miRNA with Ago2, postulated role in unwinding siRNA and miRNA/miRNA* duplexes.	(16)
	FMRP (fragile X mental retardation protein)	RNA-binding protein involved in mRNA transport and translation, etiologic factor of the fragile X syndrome.	Direct interaction, negatively modulated by FMRP phosphorylation.	Takes over RNA products from Dicer.	(17)
	Pol II (RNA polymerase II)	Catalyzes transcription of DNA to synthesize precursors of mRNAs and most snRNAs and miRNAs.	RNA-mediated interaction.	Recruits Dicer to loci of endogenous overlapping transcription, where it can cleave dsRNA into siRNA.	(18)
	5LO (5 lipoxygenase)	The key enzyme in leukotriene biosynthesis; catalyzes oxidation of arachidonic acid to hydroperoxyeicosatetraenoic acids (5-HpETE), and converts 5-HpETE to leukotriene A4.	Direct interaction with Dicer C-terminus.	Presumably influences Dicer cleavage pattern of pre-miRNAs, favoring production of ~55-nt and ~10 to ~12-nt long RNA species.	(19,20)
PROTEINS INVOLVED IN DICER TRANSPORT AND CONFERRING DICER STABILITY	CLIMP-63 (cytoskeleton-linking membrane protein 63 kDa, also known as p63)	Transmembrane protein of the endoplasmic reticulum (ER); mediates interaction between the ER and the cytoskeleton; plays a structural role for the ER morphology.	Direct interaction between N-terminal fragment of Dicer (242-430 aa) and a luminal domain of CLIMP-63.	Stabilizes newly synthesized Dicer, assists transition of Dicer through the ER and supports cellular localization of the protein (e.g., by anchoring Dicer to the nuclear periphery or in close proximity to the ribosomes on the ER).	(21)
	NDP52 (nuclear dot protein 52 kDa , also known as CALCOCO2 – calcium binding and coiled-coil domain 2)	The autophagy receptor; binds cytosolic substrates and interacts with autophagosome membrane-protein Atg8.	No data.	Mediates Dicer degradation in its RNA-free state.	(22)

CATEGORY	PROTEIN	CELLULAR ROLE	NATURE OF THE INTERACTION	RELEVANCE TO DICER	REF.
	NUP153 (nucleoporin 153)	The nuclear pore complex (NPC) protein; regulates the movement of macromolecules between the nucleus and cytoplasm.	Direct interaction.	Assists in shuttling Dicer to the NPC. Dicer associates with mobile NUP153 protein in the cytoplasm, and on the periphery of the NPC but not inside the nucleus.	(23)
VIRAL SUPPRESSORS OF SILENCING	HCV core protein	Structural protein of the viral capsid.	Direct interaction, possibly involving the helicase domain of Dicer and the N-terminal portion of the viral protein.	Inhibits Dicer processing activity.	(24,25)
	HIV-1 Tat (HIV-1 transactivator of transcription)	Plays a pivotal role in HIV-1 replication; activates transcription from the viral long terminal repeat (LTR) promoter by binding to the TAR hairpin in the nascent RNA transcript.	RNA-mediated interaction, involving helicase domain of Dicer (585-1913 aa).	Inhibits Dicer processing activity through direct interaction with Dicer and sequestration of TRBP.	(26,27)
	HIV-1 protein R (Vpr)	Plays a critical role in virus replication in non-dividing cells, induces G2 cell cycle arrest and apoptosis in proliferating cells.	Direct interaction.	Mediates proteasomal degradation of Dicer through the ubiquitin-ligase complex.	(28)

REFERENCES (SUPP. TABLE S1)

1. Chendrimada, T.P., Gregory, R.I., Kumaraswamy, E., Norman, J., Cooch, N., Nishikura, K. and Shiekhattar, R. (2005) TRBP recruits the Dicer complex to Ago2 for microRNA processing and gene silencing. *Nature*, **436**, 740-744.
2. Tahbaz, N., Kolb, F.A., Zhang, H., Jaronczyk, K., Filipowicz, W. and Hobman, T.C. (2004) Characterization of the interactions between mammalian PAZ PIWI domain proteins and Dicer. *EMBO Rep*, **5**, 189-194.
3. Lee, H.Y., Zhou, K., Smith, A.M., Noland, C.L. and Doudna, J.A. (2013) Differential roles of human Dicer-binding proteins TRBP and PACT in small RNA processing. *Nucleic Acids Res*, **41**, 6568-6576.

4. Fukunaga, R., Han, B.W., Hung, J.H., Xu, J., Weng, Z. and Zamore, P.D. (2012) Dicer partner proteins tune the length of mature miRNAs in flies and mammals. *Cell*, **151**, 533-546.
5. Lee, Y., Hur, I., Park, S.Y., Kim, Y.K., Suh, M.R. and Kim, V.N. (2006) The role of PACT in the RNA silencing pathway. *EMBO J*, **25**, 522-532.
6. Daniels, S.M., Melendez-Pena, C.E., Scarborough, R.J., Daher, A., Christensen, H.S., El Far, M., Purcell, D.F., Laine, S. and Gatignol, A. (2009) Characterization of the TRBP domain required for dicer interaction and function in RNA interference. *BMC Mol Biol*, **10**, 38.
7. Lee, H.Y. and Doudna, J.A. (2012) TRBP alters human precursor microRNA processing in vitro. *RNA*, **18**, 2012-2019.
8. MacRae, I.J., Ma, E., Zhou, M., Robinson, C.V. and Doudna, J.A. (2008) In vitro reconstitution of the human RISC-loading complex. *Proc Natl Acad Sci U S A*, **105**, 512-517.
9. Wang, H.W., Noland, C., Siridechadilok, B., Taylor, D.W., Ma, E., Felderer, K., Doudna, J.A. and Nogales, E. (2009) Structural insights into RNA processing by the human RISC-loading complex. *Nat Struct Mol Biol*, **16**, 1148-1153.
10. Haase, A.D., Jaskiewicz, L., Zhang, H., Laine, S., Sack, R., Gatignol, A. and Filipowicz, W. (2005) TRBP, a regulator of cellular PKR and HIV-1 virus expression, interacts with Dicer and functions in RNA silencing. *EMBO Rep*, **6**, 961-967.
11. Melo, S.A., Ropero, S., Moutinho, C., Aaltonen, L.A., Yamamoto, H., Calin, G.A., Rossi, S., Fernandez, A.F., Carneiro, F., Oliveira, C. *et al.* (2009) A TARBP2 mutation in human cancer impairs microRNA processing and DICER1 function. *Nat Genet*, **41**, 365-370.
12. Ota, H., Sakurai, M., Gupta, R., Valente, L., Wulff, B.E., Ariyoshi, K., Iizasa, H., Davuluri, R.V. and Nishikura, K. (2013) ADAR1 forms a complex with Dicer to promote microRNA processing and RNA-induced gene silencing. *Cell*, **153**, 575-589.
13. Ghodgaonkar, M.M., Shah, R.G., Kandan-Kulangara, F., Affar, E.B., Qi, H.H., Wiemer, E. and Shah, G.M. (2009) Abrogation of DNA vector-based RNAi during apoptosis in mammalian cells due to caspase-mediated cleavage and inactivation of Dicer-1. *Cell Death Differ*, **16**, 858-868.
14. Matskevich, A.A. and Moelling, K. (2008) Stimuli-dependent cleavage of Dicer during apoptosis. *Biochem J*, **412**, 527-534.
15. Nakagawa, A., Shi, Y., Kage-Nakadai, E., Mitani, S. and Xue, D. (2010) Caspase-dependent conversion of Dicer ribonuclease into a death-promoting deoxyribonuclease. *Science*, **328**, 327-334.
16. Robb, G.B. and Rana, T.M. (2007) RNA helicase A interacts with RISC in human cells and functions in RISC loading. *Mol Cell*, **26**, 523-537.
17. Plante, I., Davidovic, L., Ouellet, D.L., Gobeil, L.A., Tremblay, S., Khandjian, E.W. and Provost, P. (2006) Dicer-derived microRNAs are utilized by the fragile X mental retardation protein for assembly on target RNAs. *J Biomed Biotechnol*, **2006**, 64347.
18. White, E., Schlackow, M., Kamieniarz-Gdula, K., Proudfoot, N.J. and Gullerova, M. (2014) Human nuclear Dicer restricts the deleterious accumulation of endogenous double-stranded RNA. *Nat Struct Mol Biol*, **21**, 552-559.
19. Dincbas-Renqvist, V., Pepin, G., Rakonjac, M., Plante, I., Ouellet, D.L., Hermansson, A., Goulet, I., Doucet, J., Samuelsson, B., Radmark, O. *et al.* (2009) Human Dicer C-terminus functions as a 5-lipoxygenase binding domain. *Biochim Biophys Acta*, **1789**, 99-108.
20. Radmark, O., Werz, O., Steinhilber, D. and Samuelsson, B. (2007) 5-Lipoxygenase: regulation of expression and enzyme activity. *Trends Biochem Sci*, **32**, 332-341.
21. Pepin, G., Perron, M.P. and Provost, P. (2012) Regulation of human Dicer by the resident ER membrane protein CLIMP-63. *Nucleic Acids Res*, **40**, 11603-11617.
22. Gibbings, D., Mostowy, S., Jay, F., Schwab, Y., Cossart, P. and Voinnet, O. (2012) Selective autophagy degrades DICER and AGO2 and regulates miRNA activity. *Nat Cell Biol*, **14**, 1314-1321.

23. Ando, Y., Tomaru, Y., Morinaga, A., Burroughs, A.M., Kawaji, H., Kubosaki, A., Kimura, R., Tagata, M., Ino, Y., Hirano, H. *et al.* (2011) Nuclear pore complex protein mediated nuclear localization of dicer protein in human cells. *PLoS One*, **6**, e23385.
24. Wang, Y., Kato, N., Jazag, A., Dharel, N., Otsuka, M., Taniguchi, H., Kawabe, T. and Omata, M. (2006) Hepatitis C virus core protein is a potent inhibitor of RNA silencing-based antiviral response. *Gastroenterology*, **130**, 883-892.
25. Chen, W., Zhang, Z., Chen, J., Zhang, J., Wu, Y., Huang, Y., Cai, X. and Huang, A. (2008) HCV core protein interacts with Dicer to antagonize RNA silencing. *Virus Res*, **133**, 250-258.
26. Bennasser, Y. and Jeang, K.T. (2006) HIV-1 Tat interaction with Dicer: requirement for RNA. *Retrovirology*, **3**, 95.
27. Bennasser, Y., Yeung, M.L. and Jeang, K.T. (2006) HIV-1 TAR RNA subverts RNA interference in transfected cells through sequestration of TAR RNA-binding protein, TRBP. *J Biol Chem*, **281**, 27674-27678.
28. Casey Klockow, L., Sharifi, H.J., Wen, X., Flagg, M., Furuya, A.K., Nekorchuk, M. and de Noronha, C.M. (2013) The HIV-1 protein Vpr targets the endoribonuclease Dicer for proteasomal degradation to boost macrophage infection. *Virology*, **444**, 191-202.

Supplementary Table S2. Proteins influencing Dicer activity through the interactions with pre-miRNAs

TARGETED REGION OF pre-miRNA	PROTEIN NAME	CELLULAR FUNCTION	EFFECT ON DICER ACTIVITY	REF.
5′-END	BCDIN3D (BCDIN3 domain containing)	O-methyltransferase that specifically dimethylates the 5′ monophosphate of pre-miRNAs.	Negatively regulates Dicer processing, as 5′ monophosphate of pre-miRNAs is recognized by Dicer and is required for proper pre-miRNA processing.	(1)
LOOP	KSRP (KH-type splicing regulatory protein)	RNA-binding protein implicated in transcription, alternative pre-mRNA splicing, decay of labile mRNAs and certain miRNAs maturation; a part of Drosha and Dicer complex in cultured cells; binds specifically to 5′guanosine-rich motifs within the loop region of several miRNA precursors.	Promotes pre-miRNA processing by Dicer possibly by optimization of positioning and/or recruitment of miRNA-generating complexes.	(2-6)
	Lin28A	RNA-binding protein that increases the efficiency of protein synthesis by stabilizing mRNAs and driving them to polysomes; a specific suppressor of biogenesis of certain miRNAs, recognizes the tetra-nucleotide sequence motif (GGAG) in pre-miRNA apical loop; recruits terminal uridylyltransferases 4/7 (TUTase4/7).	Antagonizes Dicer processing of the certain group of pre-miRNAs by direct binding to precursors, and triggering their uridynylation and degradation.	(7-16)
	MCPIP-1 (monocyte chemoattractant protein-induced protein 1)	RNase involved in the modulations of the inflammatory response and immune homeostasis; regulates migration and infiltration of macrophages; triggers apoptosis and promotes angiogenesis.	Antagonizes Dicer processing; cleaves the apical loops of pre-miRNAs thus leading to the rapid degradation of precursors.	(17)
	RBM3 (RNA binding motif protein 3)	Cold-inducible mRNA binding protein that enhances global protein synthesis in mild hypothermic temperatures.	Positively regulates Dicer activity; binds directly to pre-miRNAs and facilitates/de-represses their ability to associate with Dicer.	(18)

TARGETED REGION OF pre-miRNA	PROTEIN NAME	CELLULAR FUNCTION	EFFECT ON DICER ACTIVITY	REF.
	TDP-43 (TAR DNA-binding protein-43)	Transcriptional repressor, splicing factor and translational regulation involved in metabolism of various RNAs; component of Drosha and Dicer complexes.	Positively regulates Dicer activity; facilitates binding of the Dicer complex to a subset of pre-miRNAs and promotes the cleavage of the specific pre-miRNAs.	(19)
3′-END	TUTase2 (3’ terminal uridylyl transferase 2), also known as PAPD4	Non-canonical, cytoplasmic poly(A) RNA polymerase.	Positively regulates Dicer processing of certain group of pre-miRNAs by monouridylation of RNA and restoring pre-miRNA’s 2-nt 3′ overhang.	(20)
	TUTase4 (3’ terminal uridylyl transferase 2), also known as ZCCHC11	3′ uridylyltransferase that acts on certain pre-miRNAs and miRNAs.	Negatively regulates Dicer processing of pre-let-7 by Lin28A-dependent polyuridylation. Positively regulates Dicer processing of a certain group of pre-miRNAs by monouridylation of RNA and restoring pre-miRNA’s 2-nt 3′ overhang.	(7,12,21)
	TUTase7 (3’ terminal uridylyl transferase 2), also known as ZCCHC6	3′ uridylyltransferase that acts on certain pre-miRNAs.	Negatively regulates Dicer processing of pre-let-7 by Lin28A-dependent polyuridylation. Positively regulates Dicer processing of a certain group of pre-miRNAs by monouridylation of RNA and restoring pre-miRNA’s 2-nt 3′ overhang.	(12,20)

REFERENCES (SUPP. TABLE S2)

1. Xhemalce, B., Robson, S.C. and Kouzarides, T. (2012) Human RNA methyltransferase BCDIN3D regulates microRNA processing. *Cell*, **151**, 278-288.
2. Nicastro, G., Garcia-Mayoral, M.F., Hollingworth, D., Kelly, G., Martin, S.R., Briata, P., Gherzi, R. and Ramos, A. (2012) Noncanonical G recognition mediates KSRP regulation of let-7 biogenesis. *Nat Struct Mol Biol*, **19**, 1282-1286.
3. Trabucchi, M., Briata, P., Garcia-Mayoral, M., Haase, A.D., Filipowicz, W., Ramos, A., Gherzi, R. and Rosenfeld, M.G. (2009) The RNA-binding protein KSRP promotes the biogenesis of a subset of microRNAs. *Nature*, **459**, 1010-1014.
4. Briata, P., Lin, W.J., Giovarelli, M., Pasero, M., Chou, C.F., Trabucchi, M., Rosenfeld, M.G., Chen, C.Y. and Gherzi, R. (2012) PI3K/AKT signaling determines a dynamic switch between distinct KSRP functions favoring skeletal myogenesis. *Cell Death Differ*, **19**, 478-487.
5. Ruggiero, T., Trabucchi, M., De Santa, F., Zupo, S., Harfe, B.D., McManus, M.T., Rosenfeld, M.G., Briata, P. and Gherzi, R. (2009) LPS induces KH-type splicing regulatory protein-dependent processing of microRNA-155 precursors in macrophages. *FASEB J*, **23**, 2898-2908.
6. Zhang, X., Wan, G., Berger, F.G., He, X. and Lu, X. (2011) The ATM kinase induces microRNA biogenesis in the DNA damage response. *Mol Cell*, **41**, 371-383.
7. Hagan, J.P., Piskounova, E. and Gregory, R.I. (2009) Lin28 recruits the TUTase Zcchc11 to inhibit let-7 maturation in mouse embryonic stem cells. *Nat Struct Mol Biol*, **16**, 1021-1025.
8. Heo, I., Joo, C., Cho, J., Ha, M., Han, J. and Kim, V.N. (2008) Lin28 mediates the terminal uridylation of let-7 precursor MicroRNA. *Mol Cell*, **32**, 276-284.
9. Heo, I., Joo, C., Kim, Y.K., Ha, M., Yoon, M.J., Cho, J., Yeom, K.H., Han, J. and Kim, V.N. (2009) TUT4 in concert with Lin28 suppresses microRNA biogenesis through pre-microRNA uridylation. *Cell*, **138**, 696-708.
10. Piskounova, E., Polytarchou, C., Thornton, J.E., LaPierre, R.J., Pothoulakis, C., Hagan, J.P., Iliopoulos, D. and Gregory, R.I. (2011) Lin28A and Lin28B inhibit let-7 microRNA biogenesis by distinct mechanisms. *Cell*, **147**, 1066-1079.
11. Piskounova, E., Viswanathan, S.R., Janas, M., LaPierre, R.J., Daley, G.Q., Sliz, P. and Gregory, R.I. (2008) Determinants of microRNA processing inhibition by the developmentally regulated RNA-binding protein Lin28. *J Biol Chem*, **283**, 21310-21314.
12. Thornton, J.E., Chang, H.M., Piskounova, E. and Gregory, R.I. (2012) Lin28-mediated control of let-7 microRNA expression by alternative TUTases Zcchc11 (TUT4) and Zcchc6 (TUT7). *RNA*, **18**, 1875-1885.
13. Viswanathan, S.R., Daley, G.Q. and Gregory, R.I. (2008) Selective blockade of microRNA processing by Lin28. *Science*, **320**, 97-100.
14. Viswanathan, S.R. and Daley, G.Q. (2010) Lin28: A microRNA regulator with a macro role. *Cell*, **140**, 445-449.
15. Newman, M.A., Mani, V. and Hammond, S.M. (2011) Deep sequencing of microRNA precursors reveals extensive 3' end modification. *RNA*, **17**, 1795-1803.
16. Newman, M.A., Thomson, J.M. and Hammond, S.M. (2008) Lin-28 interaction with the Let-7 precursor loop mediates regulated microRNA processing. *RNA*, **14**, 1539-1549.
17. Suzuki, H.I., Arase, M., Matsuyama, H., Choi, Y.L., Ueno, T., Mano, H., Sugimoto, K. and Miyazono, K. (2011) MCPIP1 ribonuclease antagonizes dicer and terminates microRNA biogenesis through precursor microRNA degradation. *Mol Cell*, **44**, 424-436.

18. Pilotte, J., Dupont-Versteegden, E.E. and Vanderklish, P.W. (2011) Widespread regulation of miRNA biogenesis at the Dicer step by the cold-inducible RNA-binding protein, RBM3. *PLoS One*, **6**, e28446.
19. Kawahara, Y. and Mieda-Sato, A. (2012) TDP-43 promotes microRNA biogenesis as a component of the Drosha and Dicer complexes. *Proc Natl Acad Sci U S A*, **109**, 3347-3352.
20. Heo, I., Ha, M., Lim, J., Yoon, M.J., Park, J.E., Kwon, S.C., Chang, H. and Kim, V.N. (2012) Mono-uridylation of pre-microRNA as a key step in the biogenesis of group II let-7 microRNAs. *Cell*, **151**, 521-532.
21. Lehrbach, N.J., Armisen, J., Lightfoot, H.L., Murfitt, K.J., Bugaut, A., Balasubramanian, S. and Miska, E.A. (2009) LIN-28 and the poly(U) polymerase PUP-2 regulate let-7 microRNA processing in Caenorhabditis elegans. *Nat Struct Mol Biol*, **16**, 1016-1020.

Supplementary Table S3. The Dicer mRNA/protein expression level in different human cancer tissues

ORGAN OR TISSUE		TYPE OF CANCER		EXPRESSION LEVEL		REF.
				mRNA	protein	
Nasopharynx		Nasopharyngeal carcinoma		↓	↓	(1)
Lungs		Non-small-cell lung carcinoma	bronchioloalveolar carcinoma and atypical adenomatous hyperplasia	↑	↑	(2)
			advanced adenocarcinoma	~	↑	(2)
			shorter postoperative survival	↓	N/A	(3)
Stomach		Gastric cancer		↓	↓	(4)
Colon or rectum		Colorectal adenocarcinoma		N/A	↑	(5)
Bladder		Urothelial carcinoma		↑	N/A	(6)
Smooth muscle		Leiomyosarcoma and leiomyoma		N/A	↑	(7)
Blood or bone marrow		Primary cutaneous T-cell lymphoma		N/A	↑	(8)
		Acute myeloid leukemia		↑	N/A	(9)
Breast		Malignant breast cancer		N/A	↓	(10)
				↓	NC	(11)
		Lymph node metastases		N/A	↑	(12)
		Aggressive basal-like, HER2+ and luminal B type tumors in breast cancer		↓	N/A	(13)
Ovaries		Ovarian carcinoma		↓	N/A	(14)
		Ovarian serous adenocarcinoma		↑	↑	(15)
		Metastatic ovarian carcinoma		↑	↑	(16)
		Invasive epithelial ovarian cancer associated with advancer tumor stage		↓	↓	(17)
Uterus		Endometrial adenocarcinoma		↓	N/A	(18)
Prostate		Prostate adenocarcinoma and prostatic intraepithelial neoplasia		N/A	↑	(19)
		Metastatic prostate adenocarcinoma		↑	N/A	
Skin and epidermis	Melanocytes	Cutaneous and acrolentiginous melanoma		N/A	↑	(20)
	Basal cells	Basal cell carcinoma		↓	N/A	(21)
	Squamous cells	Squamous cell carcinoma		↑	N/A	

N/A – not applicable, NC – no correlation, ~ similar to normal tissue, ↑ upregulated, ↓ downregulated

REFERENCES (SUPP. TABLE S3)

1. Guo, X., Liao, Q., Chen, P., Li, X., Xiong, W., Ma, J., Luo, Z., Tang, H., Deng, M., Zheng, Y. *et al.* (2012) The microRNA-processing enzymes: Drosha and Dicer can predict prognosis of nasopharyngeal carcinoma. *J Cancer Res Clin Oncol*, **138**, 49-56.
2. Chiosea, S., Jelezcova, E., Chandran, U., Luo, J., Mantha, G., Sobol, R.W. and Dacic, S. (2007) Overexpression of Dicer in precursor lesions of lung adenocarcinoma. *Cancer Res*, **67**, 2345-2350.
3. Karube, Y., Tanaka, H., Osada, H., Tomida, S., Tatematsu, Y., Yanagisawa, K., Yatabe, Y., Takamizawa, J., Miyoshi, S., Mitsudomi, T. *et al.* (2005) Reduced expression of Dicer associated with poor prognosis in lung cancer patients. *Cancer Sci*, **96**, 111-115.
4. Zheng, Z.H., Sun, X.J., Fu, W.N., Guan, Y., Gao, F., Wang, Y. and Sun, K.L. (2007) Decreased expression of DICER1 in gastric cancer. *Chin Med J (Engl)*, **120**, 2099-2104.
5. Faber, C., Horst, D., Hlubek, F. and Kirchner, T. (2011) Overexpression of Dicer predicts poor survival in colorectal cancer. *Eur J Cancer*, **47**, 1414-1419.
6. Catto, J.W., Miah, S., Owen, H.C., Bryant, H., Myers, K., Dudziec, E., Larre, S., Milo, M., Rehman, I., Rosario, D.J. *et al.* (2009) Distinct microRNA alterations characterize high- and low-grade bladder cancer. *Cancer Res*, **69**, 8472-8481.
7. Papachristou, D.J., Sklirou, E., Corradi, D., Grassani, C., Kontogeorgakos, V. and Rao, U.N. (2012) Immunohistochemical analysis of the endoribonucleases Drosha, Dicer and Ago2 in smooth muscle tumours of soft tissues. *Histopathology*, **60**, E28-36.
8. Valencak, J., Schmid, K., Trautinger, F., Wallnofer, W., Muellauer, L., Soleiman, A., Knobler, R., Haitel, A., Pehamberger, H. and Raderer, M. (2011) High expression of Dicer reveals a negative prognostic influence in certain subtypes of primary cutaneous T cell lymphomas. *J Dermatol Sci*, **64**, 185-190.
9. Martin, M.G., Payton, J.E. and Link, D.C. (2009) Dicer and outcomes in patients with acute myeloid leukemia (AML). *Leuk Res*, **33**, e127.
10. Khoshnaw, S.M., Rakha, E.A., Abdel-Fatah, T.M., Nolan, C.C., Hodi, Z., Macmillan, D.R., Ellis, I.O. and Green, A.R. (2012) Loss of Dicer expression is associated with breast cancer progression and recurrence. *Breast Cancer Res Treat*, **135**, 403-413.
11. Grelier, G., Voirin, N., Ay, A.S., Cox, D.G., Chabaud, S., Treilleux, I., Leon-Goddard, S., Rimokh, R., Mikaelian, I., Venoux, C. *et al.* (2009) Prognostic value of Dicer expression in human breast cancers and association with the mesenchymal phenotype. *Br J Cancer*, **101**, 673-683.
12. Caffrey, E., Ingoldsby, H., Wall, D., Webber, M., Dinneen, K., Murillo, L.S., Inderhaug, C., Newell, J., Gupta, S. and Callagy, G. (2013) Prognostic significance of deregulated dicer expression in breast cancer. *PLoS One*, **8**, e83724.
13. Blenkiron, C., Goldstein, L.D., Thorne, N.P., Spiteri, I., Chin, S.F., Dunning, M.J., Barbosa-Morais, N.L., Teschendorff, A.E., Green, A.R., Ellis, I.O. *et al.* (2007) MicroRNA expression profiling of human breast cancer identifies new markers of tumor subtype. *Genome Biol*, **8**, R214.
14. Pampalakis, G., Diamandis, E.P., Katsaros, D. and Sotiropoulou, G. (2010) Down-regulation of dicer expression in ovarian cancer tissues. *Clin Biochem*, **43**, 324-327.
15. Flavin, R.J., Smyth, P.C., Finn, S.P., Laios, A., O'Toole, S.A., Barrett, C., Ring, M., Denning, K.M., Li, J., Aherne, S.T. *et al.* (2008) Altered eIF6 and Dicer expression is associated with clinicopathological features in ovarian serous carcinoma patients. *Mod Pathol*, **21**, 676-684.
16. Vaksman, O., Hetland, T.E., Trope, C.G., Reich, R. and Davidson, B. (2012) Argonaute, Dicer, and Drosha are up-regulated along tumor progression in serous ovarian carcinoma. *Hum Pathol*, **43**, 2062-2069.

17. Merritt, W.M., Lin, Y.G., Han, L.Y., Kamat, A.A., Spannuth, W.A., Schmandt, R., Urbauer, D., Pennacchio, L.A., Cheng, J.F., Nick, A.M. *et al.* (2008) Dicer, Drosha, and outcomes in patients with ovarian cancer. *N Engl J Med*, **359**, 2641-2650.
18. Zighelboim, I., Reinhart, A.J., Gao, F., Schmidt, A.P., Mutch, D.G., Thaker, P.H. and Goodfellow, P.J. (2011) DICER1 expression and outcomes in endometrioid endometrial adenocarcinoma. *Cancer*, **117**, 1446-1453.
19. Chiosea, S., Jelezcova, E., Chandran, U., Acquafondata, M., McHale, T., Sobol, R.W. and Dhir, R. (2006) Up-regulation of dicer, a component of the MicroRNA machinery, in prostate adenocarcinoma. *Am J Pathol*, **169**, 1812-1820.
20. Ma, Z., Swede, H., Cassarino, D., Fleming, E., Fire, A. and Dadras, S.S. (2011) Up-regulated Dicer expression in patients with cutaneous melanoma. *PLoS One*, **6**, e20494.
21. Sand, M., Gambichler, T., Skrygan, M., Sand, D., Scola, N., Altmeyer, P. and Bechara, F.G. (2010) Expression levels of the microRNA processing enzymes Drosha and dicer in epithelial skin cancer. *Cancer Invest*, **28**, 649-653.

Supplementary Table S4. The somatic mutations in *DICER1* occurring in different types of tumor

SOMATIC MUTATION	AMINO-ACID RESIDUE AFFECTED AND ITS LOCALIZATION IN DICER		TUMOR TYPE	REF.
1304C>T	P453L	Helicase<br>C-terminal domain	GLOW	(1)
3237_3238insCCAGCAT	V1080Pfs	Ruler domain	SLCT	(2)
4031C>T	S1344L	RNase IIIa	WT	(3)
5113G>A	E1705K[1, 2]	RNase IIIb	SLCT<br>unclassified SCST<br>THC	(2)<br>(4)<br>(5)
5125G>A	D1709N[1, 2]	RNase IIIb	SLCT, PGCT (YSC)<br>SLCT<br>PPB<br>PiB	(2)<br>(4)<br>(6)<br>(7)
5125G>T	D1907Y[1,2]	RNase IIIb	PiB	(7)
5126A>G	D1709G[1, 2]	RNase IIIb	SLCT, JGCT	(2)
5127T>A	D1709E[1, 2]	RNase IIIb	SLCT, PGCT (YSC)	(2)
5138A>C	D1713A[2]	RNase IIIb	WT	(3)
5174G>A	R1725Q[2]	RNase IIIb	TGCT (seminoma)	(8)
5425G>A	G1809R	RNase IIIb	PPB	(6,9)
5425G>T	G1809W	RNase IIIb	PiB	(7)
5428G>C	D1810H[1]	RNase IIIb	SLCT	(2)
5428G>T	D1810Y[1]	RNase IIIb	SLCT, TE<br>YSC, immature TE<br>PPB	(2)<br>(4)<br>(6)
5428G>A	D1810N[1]	RNase IIIb	SLCT	(2)
5429A>G	E1788fs[3]	RNase IIIb	WT<br>Mixed GBE, DGE	(3)<br>(4)
5429A>T	D1810V[1]	RNase IIIb	SLCT with components of JGCT	(4)
5437G>C	E1813Q[1]	RNase IIIb	SLCT	(2,4)
5437G>A	E1813K[1]	RNase IIIb	SLCT<br>cERMS<br>PiB	(2,4)<br>(10)<br>(7)
5438A>G	E1813G[1]<br><br>E1788fs[3]	RNase IIIb	SLCT<br>PPB<br>THC<br>WT<br>YSC, SLCT, DGE, eCA, immature TE, CHC	(2)<br>(6,11)<br>(5)<br>(3)<br>(4)
5438A>T	E1813V[1]	RNase IIIb	PiB	(12)<br>(7)
5438A>C	E1813A[1]	RNase IIIb	NHL (dlBCL)	(13)
5439G>T<br><br>5439G>C	E1813D[1]	RNase IIIb<br><br>RNase IIIb	PiB<br>THC<br>SLCT	(7)<br>(5)<br>(4)
5452G>A	A1818T	RNase IIIb	WT	(3)
5492G>A	W1831X	RNase IIIb	PGCT (YST)	(2)
5529T>C	R1898G	dsRBD	GLOW	(1)

Mutations in the RNase IIIb domain: [1]metal-binding site affected; [2]conserved region affected; [3]additional exonic silencing site generated, predicted to exon 25 skipping and the expression of Dicer lacking the majority of its RNase IIIb domain.

Abbreviations: cERMS – cervical embryonal rhabdomyosarcoma, CHC – choriocarcinoma, DGE – dysgerminoma, eCA – embryonal carcinoma, fs – frame-shift of the open reading frame, GBE – gonadoblastoma, GLOW - tumors associated with GLOW syndrome, JGCT – juvenile granulosa cell tumor, NHL (dlBCL) – non-Hodgkin lymphoma (diffuse large B-cell lymphoma), PiB- pituitary blastoma, PGCT – primitive germ-cell tumor, PPB – pleuropulmonary blastoma, SCST – sex cord-stromal tumor, SLCT – Sertoli-Leydig cell tumor, TE- teratoma, TGCT – testicular germ cell tumor, THC – thyroid carcinoma, WT – Wilms tumor, YSC – yolk sac tumor

REFERENCES (SUPP. TABLE S4)

1. Klein, S., Lee, H., Ghahremani, S., Kempert, P., Ischander, M., Teitell, M.A., Nelson, S.F. and Martinez-Agosto, J.A. (2014) Expanding the phenotype of mutations in DICER1: mosaic missense mutations in the RNase IIIb domain of DICER1 cause GLOW syndrome. *J Med Genet*, **51**, 294-302.
2. Heravi-Moussavi, A., Anglesio, M.S., Cheng, S.W., Senz, J., Yang, W., Prentice, L., Fejes, A.P., Chow, C., Tone, A., Kalloger, S.E. *et al.* (2012) Recurrent somatic DICER1 mutations in nonepithelial ovarian cancers. *N Engl J Med*, **366**, 234-242.
3. Wu, M.K., Sabbaghian, N., Xu, B., Addidou-Kalucki, S., Bernard, C., Zou, D., Reeve, A.E., Eccles, M.R., Cole, C., Choong, C.S. *et al.* (2013) Biallelic DICER1 mutations occur in Wilms tumours. *J Pathol*, **230**, 154-164.
4. Witkowski, L., Mattina, J., Schonberger, S., Murray, M.J., Choong, C.S., Huntsman, D.G., Reis-Filho, J.S., McCluggage, W.G., Nicholson, J.C., Coleman, N. *et al.* (2013) DICER1 hotspot mutations in non-epithelial gonadal tumours. *Br J Cancer*, **109**, 2744-2750.
5. de Kock, L., Sabbaghian, N., Soglio, D.B., Guillerman, R.P., Park, B.K., Chami, R., Deal, C.L., Priest, J.R. and Foulkes, W.D. (2014) Exploring the association Between DICER1 mutations and differentiated thyroid carcinoma. *J Clin Endocrinol Metab*, **99**, E1072-1077.
6. Seki, M., Yoshida, K., Shiraishi, Y., Shimamura, T., Sato, Y., Nishimura, R., Okuno, Y., Chiba, K., Tanaka, H., Kato, K. *et al.* (2014) Biallelic DICER1 mutations in sporadic pleuropulmonary blastoma. *Cancer Res*, **74**, 2742-2749.
7. de Kock, L., Sabbaghian, N., Plourde, F., Srivastava, A., Weber, E., Bouron-Dal Soglio, D., Hamel, N., Choi, J.H., Park, S.H., Deal, C.L. *et al.* (2014) Pituitary blastoma: a pathognomonic feature of germ-line DICER1 mutations. *Acta Neuropathol*, **128**, 111-122.
8. de Boer, C.M., Eini, R., Gillis, A.M., Stoop, H., Looijenga, L.H. and White, S.J. (2012) DICER1 RNase IIIb domain mutations are infrequent in testicular germ cell tumours. *BMC Res Notes*, **5**, 569.
9. Murray, M.J., Bailey, S., Raby, K.L., Saini, H.K., de Kock, L., Burke, G.A., Foulkes, W.D., Enright, A.J., Coleman, N. and Tischkowitz, M. (2014) Serum levels of mature microRNAs in DICER1-mutated pleuropulmonary blastoma. *Oncogenesis*, **3**, e87.

10. Tomiak, E., de Kock, L., Grynspan, D., Ramphal, R. and Foulkes, W.D. (2014) DICER1 mutations in an adolescent with cervical embryonal rhabdomyosarcoma (cERMS). *Pediatr Blood Cancer*, **61**, 568-569.

11. de Kock, L., Plourde, F., Carter, M.T., Hamel, N., Srivastava, A., Meyn, M.S., Arseneau, J., Bouron-Dal Soglio, D. and Foulkes, W.D. (2013) Germ-line and somatic DICER1 mutations in a pleuropulmonary blastoma. *Pediatr Blood Cancer*, **60**, 2091-2092.

12. Sahakitrungruang, T., Srichomthong, C., Pornkunwilai, S., Amornfa, J., Shuangshoti, S., Kulawonganunchai, S., Suphapeetiporn, K. and Shotelersuk, V. (2014) Germline and Somatic DICER1 Mutations in a Pituitary Blastoma Causing Infantile-Onset Cushing's Disease. *J Clin Endocrinol Metab*, **99**, E1487-1492.

13. Lee, S.H., Kim, M.S. and Yoo, N.J. (2013) Mutation analysis of DICER1 gene in hematologic tumors. *Leuk Lymphoma*, **54**, 2551-2552.
